# Human Hair Follicle Mesenchymal Stem Cell-Derived Exosomes Attenuate UVB-Induced Photoaging via the miR-125b-5p/TGF-β1/Smad Axis

**DOI:** 10.34133/bmr.0121

**Published:** 2025-01-13

**Authors:** Hong Cui, Luo-Qin Fu, Yan Teng, Jun-Jia He, Ye-Yu Shen, Qiong Bian, Ting-Zhang Wang, Mei-Xia Wang, Xiang-Wei Pang, Zhi-Wei Lin, Min-Gang Zhu, Yu Cai, Yang-Yang Li, Jin-Yang Chen, Xiao-Zhou Mou, Yi-Bin Fan

**Affiliations:** ^1^ Center for Plastic & Reconstructive Surgery, Department of Dermatology, Zhejiang Provincial People’s Hospital, Affiliated People’s Hospital, Hangzhou Medical College, Hangzhou 310014, Zhejiang, China.; ^2^ Clinical Research Institute, Zhejiang Provincial People’s Hospital, Affiliated People’s Hospital, Hangzhou Medical College, Hangzhou 310014, China.; ^3^ Key Laboratory of Microbiol Technology and Bioinformatics of Zhejiang Province, Zhejiang Institute of Microbiology, Hangzhou 310012, China.; ^4^ HealthRegen (Hangzhou) Biotechnology Co., Ltd, Hangzhou 310052, China.; ^5^ Department of Dermatology, The First People’s Hospital of Jiashan, Jiaxing 314100, China.; ^6^ Women’s Hospital, Zhejiang University School of Medicine, Hangzhou 310006, China.

## Abstract

Cutaneous photoaging, induced by chronic exposure to ultraviolet (UV) radiation, typically manifests as alterations in both the physical appearance and functional properties of the skin and may predispose individuals to cancer development. Recent studies have demonstrated the reparative potential of exosomes derived from mesenchymal stem cells in addressing skin damage, while specific reports highlight their efficacy in ameliorating skin photoaging. However, the precise role of exosomes derived from human hair follicle mesenchymal stem cells (HFMSC-Exos) in the context of cutaneous photoaging remains largely unexplored. We successfully isolated HFMSC-Exos using the ultracentrifugation technique. In cellular experiments, we assessed the migration of human dermal fibroblasts (HDFs) through scratch and transwell assays, evaluated the angiogenesis of human umbilical vein endothelial cells through angiogenesis assays, and examined the expression levels of collagen and matrix metalloproteinase 1 (MMP-1) using Western blotting and quantitative reverse transcription polymerase chain reaction. Furthermore, we established a nude mouse model of photoaging to observe wrinkle formation on the dorsal surface of the animals, as well as to assess dermal thickness and collagen fiber generation through histological staining. Ultimately, we performed RNA sequencing on skin tissues from mice before and after treatment to elucidate the relevant underlying mechanisms. Our findings revealed that HFMSC-Exos effectively enhanced the migration and proliferation of HDFs and upregulated the expressions of transforming growth factor-β1 (TGF-β1), p-Smad2/p-Smad3, collagen type 1, and collagen type 3 while concurrently down-regulating MMP-1 levels in HDFs. Additionally, mice in the HFMSC-Exo group showed quicker wrinkle healing and increased collagen production. HFMSC-Exos miR-125b-5p was demonstrated to reduce skin photoaging by increasing profibrotic levels via TGF-β1 expression. UV-irradiated HDFs and photoaged nude mouse skin showed low TGF-β1 expressions, whereas overexpression of TGF-β1 in HDFs increased collagen type 1, collagen type 3, and p-Smad2/p-Smad3 expressions while decreasing MMP-1 expression. HDFs overexpressing TGF-β1 produced more collagen and altered the Smad pathway. This study demonstrated, both in vitro and in vivo, that HFMSC-Exos increased collagen formation, promoted HDF cell proliferation and migration, and reversed the senescence of UV-irradiated HDFs. TGF-β1 was identified as a target of HFMSC-Exos miR-125b-5p, which controls photoaging via regulating the Smad pathway. The antiphotoaging capabilities of HFMSC-Exos may occur via the miR-125b-5p/TGF-β1/Smad axis, suggesting a promising therapeutic approach for treating skin photoaging.

## Introduction

Skin photoaging, a chronic manifestation of skin photodamage that typically occurs following exposure to ultraviolet (UV) radiation, is characterized by deterioration of the extracellular matrix and a reduction in collagen levels [1]. A pivotal event in the progression and pathophysiology of photoaging is the degradation of collagen fibers within the extracellular matrix after UV exposure [2]. This process is marked by the upregulation of the production of matrix metalloproteinases upon UV radiation exposure, resulting in the breakdown of collagen type 1 (COL-1) and collagen type 3 (COL-3), the principal constituents of the extracellular matrix [3]. Physiological and psychological complications arise from photoaging, leading to aesthetic deterioration and dysfunction. Firstly, drug therapy is a common approach to photoaging treatment. Drug therapy usually takes a long time to show results and may have side effects or contraindications for certain populations. Secondly, physical therapy also occupies a place in photoaging treatment. These treatment methods usually have notable effects, but they are expensive and require professional doctors to perform, posing certain risks. In addition, some natural therapies and skincare products are used as adjunctive treatments for photoaging. However, the effectiveness of these products varies from person to person and requires long-term adherence to use in order to see significant results. However, the current strategies for preventing and treating photoaging are inadequate. Therefore, it is imperative that we explore novel clinical treatments.

Mesenchymal stem cell-derived exosomes (MSC-Exos) have been widely utilized as therapeutic interventions for tissue damage because of their low immunogenicity, improved biocompatibility, and wider diffusion [4]. The healing potential of MSC-Exos has been attributed to their paracrine mechanisms and capacity to heal tissues [5]. MSC-Exos secreted by mesenchymal stem cells (MSCs) and collected from MSC-conditioned medium reportedly reduced tissue damage in the skin, liver, heart, and lungs. Previous studies demonstrated that MSC-Exos enhance the biological activity of skin fibroblasts by promoting cell proliferation and reducing apoptosis [6,7]. However, the potential of human hair follicle MSC-derived exosomes (HFMSC-Exos) to slow skin photoaging has not been extensively investigated and the underlying mechanisms therein remain unclear. Exosomes serve as natural mechanisms for the transportation of genetic information between cells, including messenger RNAs (mRNAs), microRNAs (miRNAs), proteins, and lipids, thus facilitating intercellular communication. Recent studies implicated miRNA-rich exosomes in the pathophysiology of tissue regeneration [8,9]. While the impact of miR-125b-5p on photoaging disorders remains under debate, some researchers have observed that miR-125b-5p exhibits antiphotoaging properties in UVA-induced photodamage [10]. The primary focus of this investigation was to examine the role of miR-125b-5p derived from HFMSC-Exos in skin photoaging.

Endogenous miRNAs, short noncoding RNAs 18 to 25 nucleotides long, can regulate gene expression by upregulating or enhancing the translation of specific mRNAs. MiRNAs play a direct role in skin photoaging by serving as epigenetic regulators [11]. The cloning of miR-125b-5p was initially accomplished by Zhang et al. [12] and Liao et al. [13]. A bioinformatics analysis predicted a potential complementary binding relationship between transforming growth factor-β1 (TGF-β1) 3′-untranslated region (UTR) and miR-125b-5p. TGF-β1, initially discovered in 1988 [14], has been predominantly observed in smooth muscle cells, fibroblasts, epithelial cells, and microvascular endothelial cells [15–17]. Although the precise mechanisms underlying its impact on skin photoaging remain incompletely understood, previous studies indicated that its overexpression or augmentation can enhance skin healing, reduce inflammation, and offer other advantages [18,19]. Thus, given its established role as a pivotal mediator of skin photoaging-related disorders, is it feasible for TGF-β1 to regulate skin photoaging via the Smad pathway? TGF-β1 induces Smad2 and Smad3 phosphorylation. We hypothesize that the miR-125b-5p/TGF-β1/Smad axis serves as a mediator of the antiphotoaging effects of HFMSC-Exos.

In this study, we isolated human hair follicle MSCs (HFMSCs) and HFMSC-Exos, conducted in vitro and in vivo experiments to assess the impact of HFMSC-Exos on skin photoaging, and investigated the mechanisms underlying the antiphotoaging ability of HFMSC-Exos. The in vitro experiments revealed that HFMSC-Exos upregulated collagen expression and down-regulated matrix metalloproteinase 1 (MMP-1) expression in human dermal fibroblasts (HDFs) while facilitating UV-irradiated HDF migration and proliferation. In vivo studies demonstrated that HFMSC-Exos promote wrinkle recovery and augment collagen deposition in a photoaging nude mouse model. Furthermore, we present evidence suggesting that miR-125b-5p in HFMSC-Exos enhances antiphotoaging effects on skin, with miR-125b-5p targeting TGF-β1 to regulate the Smad signaling pathway involved in photoaging formation. In conclusion, our study offers a rational explanation for the therapeutic use of HFMSC-Exos to treat skin photoaging.

## Materials and Methods

### Culture of HDFs, human umbilical vein endothelial cells, and primary HFMSCs

Briefly, HDFs and human umbilical vein endothelial cells (HUVECs) were purchased from Zhejiang Mason Cell Technology (Jinhua, China) and characterized using short tandem repeat analysis. Subsequently, the cells were cultivated in a moisture-controlled incubator and maintained at 37 °C and 5% CO_2_ using high-sugar Dulbecco’s modified Eagle’s medium (Vivacell, Germany) supplemented with 10% fetal bovine serum (Serana, Germany), 100 U/ml penicillin, and 100 μg/ml streptomycin. The HDFs and HUVECs were seeded in 6-well plates at a density of 2 × 10^5^ cells/well. Once the cells reached a density of 70% to 80%, they were exposed to UVB irradiation to induce senescence. Subsequently, the HDFs were treated with HFMSC-Exos at concentration of 10 μg/ml (UVB-Exo-L) and 20 μg/ml (UVB-Exo-H) as well as Lipofectamine 3000 reagent (Life Technologies, Carlsbad, USA) transfected with miR-125b-5p mimics or TGF-β1 lentiviral vectors. These treatments were administered for approximately 24 or 48 h to assess protein and mRNA levels. Lysates were used to examine the expressions of Smad pathway markers, specifically COL-1, COL-3, and MMP-1. The HUVECs were subjected to angiogenesis experiments involving treatment with 10 and 20 μg/ml HFMSC-Exos, and the ability of HFMSC-Exos to promote angiogenesis was demonstrated as tubule generation.

Primary HFMSCs were acquired from HealthRegen (Hangzhou) Biotechnology Co., Ltd. (Hangzhou, China). The cells underwent cultivation in a moisture-controlled incubator with 5% CO_2_ at 37 °C using low-sugar Dulbecco’s modified Eagle’s medium (Vivacell) enriched with 20% fetal bovine serum (Serana), 100 U/ml penicillin, and 100 μg/ml streptomycin. To characterize the phenotypes, cells from P4-HFMSC were incubated with various fluorescent antibodies (CD90–fluorescein isothiocyanate, CD105–phycoerythrin, CD14–ECD, CD34–PC5, CD49d–fluorescein isothiocyanate, CD73–phycoerythrin, HLA-DR–ECD, and CD45–PC5) followed by flow cytometry analysis (Agilent). To induce adipogenic and osteogenic differentiation, approximately 80% to 90% confluent HFMSCs were cultivated in 6-well cultures using a 0.1% gelatin solution. Differentiation was induced by culturing in 2 different media, osteogenic and adipogenic, for 3 weeks. The cells were then fixed with 4% paraformaldehyde followed by staining with Oil Red O and Alizarin Red S to check the culturing results. Images were captured using an EVOS M7000 microscope (Invitrogen). For chondrogenic differentiation, approximately 150,000 cells were resuspended in 15-ml centrifuge tubes, centrifuged to the bottom, and induced with chondrogenic differentiation induction medium for 3 weeks. HFMSCs induced for chondrogenic differentiation were stained with Alcian blue.

### Isolation, identification, and labeling of HFMSC-Exos

In accordance with the protocol provided by EVital Bio (Hangzhou) Co., Ltd, first, HFMSCs were cultivated in 10-cm cell culture dishes. When the cells attained approximately 70% to 80% confluency, the existing culture medium was replaced with a serum-free medium for 24 to 48 h. The supernatant from the cell culture was collected, and the exosomes were separated via differential centrifugation [20]. Every step of the centrifugation process was performed at 4 °C. Initially, the supernatant was centrifuged at 300 × *g* for 10 min to remove the cells. Subsequently, the supernatant was centrifuged at 2,000 × *g* for 10 min to eliminate cellular debris and apoptotic entities. Subsequently, the supernatant was centrifuged at 10,000 × *g* for 30 min and then at 100,000 × *g* for 70 min using an ultracentrifuge (Beckman Coulter, Beverly, Massachusetts, USA) with a single repetition. HFMSC-Exos were collected and stored at −80 °C. Postcentrifugation, the exosomes were reconstituted in 50 to 200 μl of phosphate-buffered saline (PBS), their morphology was instantly observed via transmission electron microscopy, and their size distribution was examined via NanoSight tracking analysis (ZetaView system). Concurrently, immunoblotting was employed to identify the presence of the recognized exosome indicators CD9, CD63, and CD81. Immunoblotting was used to examine the expression of the exosome-negative marker calnexin. Subsequently, the concentration of the exosomal proteins was quantified using a bicinchoninic acid assay kit.

Prior to the experiment, the exosomes were diluted in a culture medium and passed through a 0.22-μm membrane to eliminate any bacteria. Purified exosomes were labeled with the red fluorescent dye DiR (Biotium) to investigate the internalization by HDFs. Specifically, 200 μl of PBS containing exosomes was incubated with an appropriate amount of the DiR dye for 5 min. Excess dye was counteracted with 1 ml of serum devoid of exosomes. Subsequently, the blend underwent ultracentrifugation at 4 °C for 70 min at 100,000 × *g* to eliminate the supernatant and gather the pellet, which was then reconstituted in PBS. DiR-labeled exosomes were coincubated with UV-irradiated HDFs for 24 h. The cells were fixed with 4% paraformaldehyde, washed thrice with PBS, and blocked with 4′,6-diamidino-2-phenylindole-containing blocking solution, and the internalized HDFs were visualized using a TCS SP8 confocal microscope (Leica, Germany).

### Grouping and treatment of in vivo and in vitro treatment experiments

HDF cells were divided into 4 groups: (a) control, (b) UVB, (c) UVB + HFMSC-Exos (10 μg/ml), and (d) UVB + HFMSC-Exos (20 μg/ml). The dosage used for extracellular vesicles is calculated based on protein concentration. In vivo experiments were divided into 4 groups: (a) control, (b) UVB, (c) UVB + daub, and (d) UVB + immunohistochemical staining. The dose of extracellular vesicles used in the in vivo experiment is 20 μg per unit time. The UVB irradiation dose for in vitro experiments is 20 mJ/cm^2^, with a duration of 30 min. The irradiation dose for the in vivo experiment is 150 mJ/cm^2^, irradiated every other day for 30 min per day for 8 consecutive weeks.

### Cell transfection

Firstly, logarithmic growth cells were seeded onto a 6-well plate, and a transfection reagent for overexpressing lentivirus and miRNA was prepared using Lipofectamine 3000. The resulting mixture was then added to the cells, and the complex entered the cells through endocytosis. Overexpression transfection was screened with antibiotics for at least 3 generations.

### Quantitative real-time polymerase chain reaction

Lysis of the samples was performed using TRIzol reagent (Invitrogen) for extraction and measurement of the total RNA concentration. Following this, 1,000 ng of RNA was reverse-transcribed into complementary DNA (cDNA) using the Prime Script reverse transcription kit (Takara, Dalian, China). The resulting cDNA was subjected to real-time quantification using an ABI 7500 Real-Time Fluorescence Quantification System with SYBR mixture (Takara, Dalian, China). The mixture underwent predenaturation at 95 °C for 10 min, followed by 40 repetitions at 95 °C for 15 s and annealing at 60 °C for 1 min, culminating in a phase of melting curves. The comparative expression levels of the target genes were ascertained through the 2^−ΔΔCT^ technique, and each reaction was replicated 3 times. For comparative measurements, the expression levels were standardized against glyceraldehyde-3-phosphate dehydrogenase.

### Western blotting

Fibroblast and skin samples were collected and washed twice with PBS to extract proteins from the cells and tissues. The cell lysis buffer (radioimmunoprecipitation assay; Beyotime, China) was supplemented with protease inhibitors (phenylmethylsulfonyl fluoride; Beyotime). The lysed specimens were incubated on ice for 30 min. Subsequently, the lysis blend underwent centrifugation at 4 °C and 14,400 rpm to remove any cellular remnants. A bicinchoninic kit was used to measure the protein concentration (Thermo Fisher). Specifically, 20 μg of total proteins was loaded onto a 10% sodium dodecyl sodium–polyacrylamide gel electrophoresis gel and subsequently transferred to a polyvinylidene difluoride transfer membrane (Millipore, Billerica, Massachusetts, USA). The gel was run at 80 V for 30 min, followed by 120 V for 30 to 60 min. The polyvinylidene difluoride membranes were then sealed with a 5% skim milk solution diluted in Tris-buffered saline with Tween-20 for 2 h at room temperature. Subsequently, the membranes underwent overnight incubation at 4 °C using these specific antibodies: COL-1 (1:1,000; ABclonal, China); COL-3 (1:1,000; Abcam, UK); MMP-1 (1:1,000; Abcam); CD9, CD63, CD81, and calnexin (1:1,000; Abcam), Smad2/3 (1:1,000; ABclonal); TGF-β1 (1:1,000; ABclonal); and β-actin (1:50,000; ABclonal). The following day, the cells were incubated with horseradish peroxidase-conjugated antirabbit immunoglobulin G secondary antibody (1:5,000; ABclonal) for 2 h at room temperature. To detect proteins through chemiluminescence, immunoreactive protein bands on the membranes were observed using a Bio-Rad system and an enhanced chemiluminescence kit (Bio-Rad). Protein expression intensities were analyzed using the ImageJ software to normalize the expression of target proteins based on internal reference proteins.

### Wound scratch and transwell migration assays

When the cells reached approximately 100% density in the 6-well plates, the medium was replaced with serum-free medium. Subsequently, the cells were exposed to UV irradiation and then scratched using a 200-μl sterile pipette tip to create cell gaps. The cells were washed 3 times with PBS and treated with HFMSC-Exos or an equivalent volume of PBS. After 24 h, an EVOS M7000 microscope was used in conjunction with the ImageJ software to observe and quantify the migration rate across the scratches.

A 24-well transwell migration system with an 8-μm filter membrane was positioned in the upper chamber of a 24-well plate. Subsequently, 100 μl of a cell suspension containing 5 × 10^4^ HDFs/chamber was introduced into the upper chamber. Next, 100 μl of a solution with HFMSC-Exos (10 or 20 μg/ml), or a similar amount of PBS, was added to the higher chamber and left to incubate for 24 h. Subsequently, HDFs were fixed with 4% paraformaldehyde for 30 min, followed by a triple rinse with PBS. Subsequently, the HDFs were stained with 0.5% crystal violet solution (Beyotime) for 30 min at ambient temperature. After the staining process was completed and the cells were rinsed with double-distilled water, the cells located in the upper chamber were carefully removed and the number of migrated cells was observed using an EVOS M7000 microscope and quantified using the ImageJ software.

### Effect of HFMSC-Exos on HDF proliferation measured by Cell Counting Kit-8

The cells were uniformly seeded in 96-well plates at a density of 8,000 to 10,000 cells/well. After plating, the cells were exposed to UV irradiation and treated with varying concentrations of HFMSC-Exos for 24 to 48 h. Ten microliters of Cell Counting Kit-8 reagent was added to each 100 μl of the mixture and incubated for 50 to 60 min. The absorbance at 450 nm was measured using an enzyme marker, and the cell proliferation rate was calculated using the formula (treatment group − blank group)/(control group − blank group).

### Effects of HFMSC-Exos on HDF senescence observed by β-galactosidase staining assay

HDFs were uniformly inoculated into 6-well plates; when the cell fusion reached 70% to 80%, UVB irradiation was performed, the cell culture medium was aspirated, the cells were washed once with PBS, 1 ml of β-galactosidase-stained fixative was added, and the cells were fixed for 15 min at room temperature. The cells were then washed 3 times with PBS, 1 ml of the staining working solution was added to each well, and the plates were incubated at 37 °C overnight. The cells were observed and photographed under an ordinary light microscope, and the cell senescence rate was calculated and quantified using the ImageJ software.

### Angiogenesis experiment

HUVECs at the logarithmic growth phase were taken and uniformly inoculated on a 6-well plate at a density of 2 × 10^5^/well. After the cells adhered to the wall, UVB irradiation was performed according to the experimental groups, and different concentrations of HFMSC-Exos or PBS were added to the coculture for 24 h. On the day before conducting the angiogenesis experiment, the matrix gel taken from the −80 °C refrigerator was placed overnight in a 4 °C refrigerator to slowly melt. Before the experiment, the gun head was cooled and a 24-hole plate was used in advance, and the entire experiment was performed on ice; 100 μl of matrix adhesive was evenly spread on each well of the 24-well plate to prevent the formation of bubbles and allowed to stand for 1 to 2 h in a 37 °C incubator to solidify. Previously treated HUVECs were taken from each group and evenly inoculated onto a 24-well plate covered with matrix adhesive at a density of 5 × 10^4^/well. This was repeated for 2 wells in each group. The sample was incubated in a 37 °C incubator, and the formation of blood vessels was observed after 4 to 6 h. An EVOS M7000 microscope was used to capture images, and the ImageJ software was used to calculate the angiogenesis rate.

### Detection of reactive oxygen species generation in HDFs by dichlorodihydrofluorescein diacetate staining

HDFs were inoculated on 35-mm Petri dishes with a 14-mm-diameter glass bottom and grown to a density of approximately 50% with or without UV irradiation. The cell culture medium was removed, an appropriate volume of diluted dichlorodihydrofluorescein diacetate (DCFH-DA) was added, and the cells were incubated for 20 min at 37 °C in a cell culture incubator. The HDFs were then washed 3 times with serum-free cell culture medium to completely remove DCFH-DA that did not enter the cells, which was directly observed using a laser confocal microscope, and the mean fluorescence intensity was calculated using the ImageJ software.

### Luciferase reporter assay

Wild-type TGF-β1 3′-UTR, mutant TGF-β1 3′-UTR, or a nontargeting control RNA was cotransfected with miR-125b-5p mimicry using Lipofectamine 3000 reagent (50 nM) as the transfection agent. After 24 h, samples were collected and subjected to a luciferase assay.

### In vivo effect of HFMSC-Exos

Five-week-old female nude mice were obtained from the Experimental Animal Center of Zhejiang Provincial People’s Hospital. The animal experimental protocols followed the guidelines set by the Experimental Animal Committee of the Zhejiang Provincial People’s Hospital. The mice were randomly segregated into 4 distinct clusters: control group, UVB irradiation-positive group, HFMSC-Exos daubed group (20 μg diluted in 100 μl of PBS), and HFMSC-Exo subcutaneous injection group (20 μg diluted in 100 μl of PBS). The mice were subjected to alternate-day UV irradiation for 8 weeks, followed by 2-week treatment with HFMSC-Exos via coating or subcutaneous injection on alternate days. Digital photographs of the dorsal skin were captured at weeks 0, 8, and 20, while the wrinkles were analyzed and quantified using the International Wrinkle Severity Rating Scale. After a 10-week interval, the mice were euthanized and dorsal skin samples were collected for subsequent histological analyses. Each experimental group included a minimum of 5 mice.

### Histopathological analysis

The specimens were stabilized using a 4% paraformaldehyde mixture, dehydrated using anhydrous ethanol, encased in paraffin, and subsequently sectioned to a 5-μm thickness. Histological changes were assessed using hematoxylin and eosin and Masson’s trichrome staining. Immunohistochemical staining was performed by first deparaffinizing the sections, followed by immersion in a 3% H_2_O_2_ solution at 37 °C for 15 min to eliminate endogenous peroxidase activity. To prevent nonspecific binding, the sections were incubated in a solution of 5% PBS diluted in bovine serum albumin for 1 h. Subsequently, the samples were incubated overnight at 4 °C using primary antibodies aimed at CD31. The next day, the cells were incubated with a secondary antibody and stained with diaminobenzidine. Image acquisition was facilitated using an EVOS M7000 microscope.

### Biochemical testing

The prepared mouse serum samples were analyzed using a fully automated biochemical analyzer to determine the levels of alanine aminotransferase, aspartate aminotransferase, blood urea nitrogen, and creatinine in mouse serum, in order to evaluate the safety of HFMSC-Exos.

### RNA isolation, cDNA library preparation, and Illumina sequencing for transcriptome analysis

Total RNA was extracted using TRIzol reagent (Invitrogen) according to the manufacturer’s protocol. The mRNA was purified from total RNA using oligo(dT) magnetic beads and fragmented into small pieces in fragmentation buffer at 37 °C for 10 min. A first-strand cDNA synthesis was performed using SuperScript II (Promega) and random primers, while a second-strand cDNA synthesis was performed using DNA polymerase I and RNase H (Promega). Double-stranded cDNA was purified using an AMPure XP system (Beckman Coulter). The purified double-stranded cDNA was end-repaired using T4 DNA polymerase (New England Biolabs), Klenow enzyme (New England Biolabs), and T4 polynucleotide kinase (New England Biolabs), followed by a single A base addition using Klenow exopolymerase and then ligated with an adapter using DNA ligase (New England Biolabs). To select cDNA fragments of 250 to 300 bp in length, the library fragments were purified using the AMPure XP system (Beckman Coulter) and amplified by polymerase chain reaction to create a cDNA library. The cDNA library products were quantified using Qubit 2.0 and then evaluated using an Agilent 2100 analyzer and qualitative polymerase chain reaction (Invitrogen). Finally, the library preparations were sequenced on an Illumina Nova 6000 platform, which generated 150-bp paired-end reads.

### Statistical analysis

All statistical evaluations were performed using the GraphPad Prism 9 software. Each experiment was replicated a minimum of 3 times, with the data presented as mean ± standard error of the mean. Independent-samples *t* tests were used to compare 2 groups, whereas analysis of variance was used for multiple comparisons. Statistical significance was set at *P* < 0.05.

## Results

### Characterization of human HFMSCs and HFMSC-Exos

**Fig. 1.**
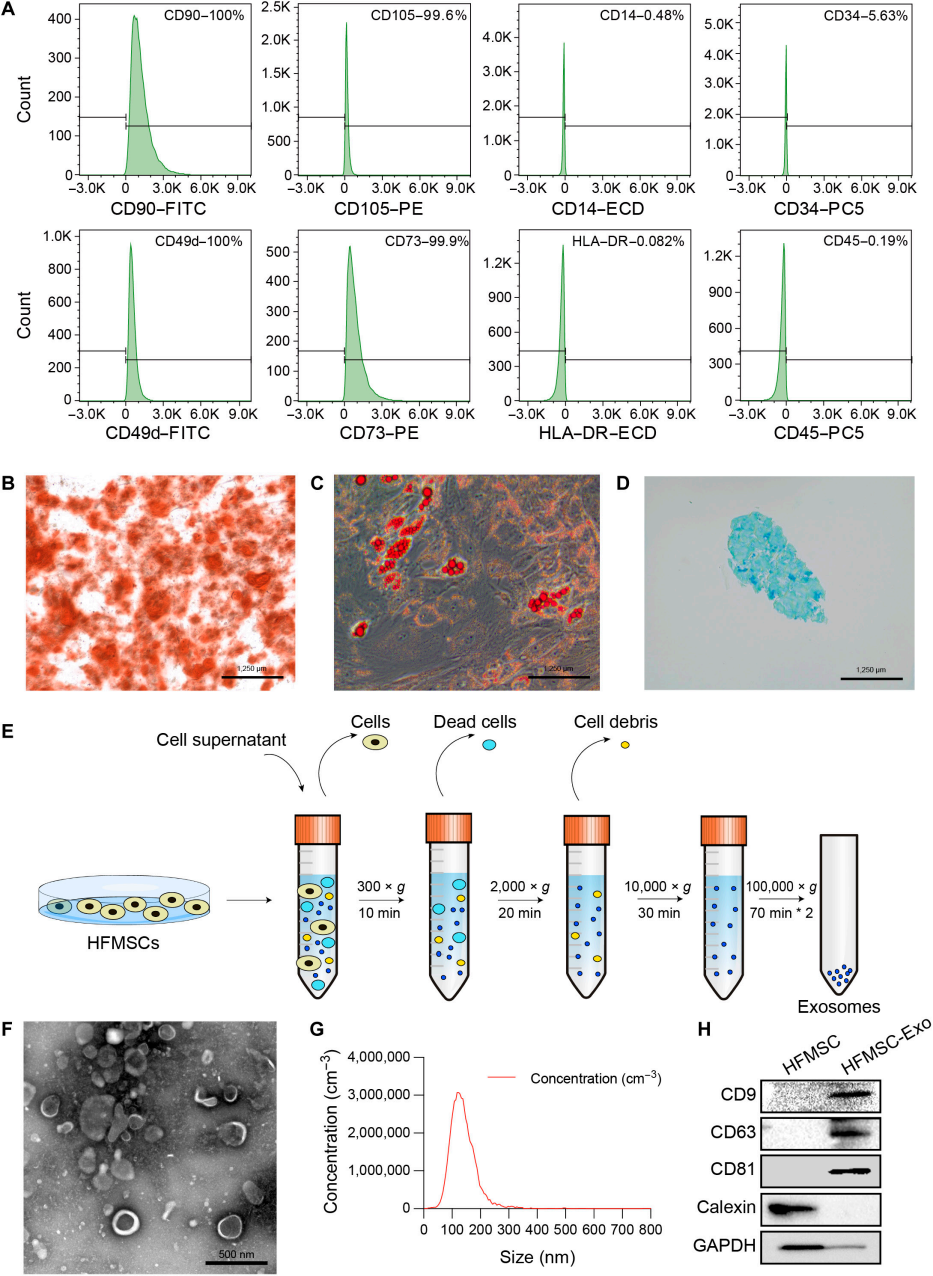
Identification of HFMSCs and HFMSC-Exo. (A) Representative flow cytometry analysis of HFMSCs, MSC surface markers (CD90, 99.8%; CD105, 99.6%; CD49d, 100%; and CD73, 99.9%) and HSC surface markers (CD14, 0.48%; CD34, 5.63%; HLA-DR, 0.048%; and CD45, 0.19%). (B) Image of osteogenic differentiation measured by Alizarin Red S staining (scale bar = 500 μm). (C) Image of adipogenic differentiation measured by Oil Red O staining (scale bar = 500 μm). (D) Image of chondrogenic differentiation measured by Alcian blue staining. (E) Schematic diagram of the HFMSC-Exo preparation process. (F) Transmission electron microscopy analysis of HFMSC-Exo morphology (scale bar = 500 nm). (G) Particle size distribution in HFMSC-Exos was quantified using NanoSight tracking analysis. (H) Western blotting examination of recognized positive indicators (CD9, CD63, and CD81) and negative indicators (calnexin). FITC, fluorescein isothiocyanate; PE, phycoerythrin; HFMSC, human hair follicle mesenchymal stem cells; HFMSC-Exos, exosomes derived from human hair follicle mesenchymal stem cells; MSCs, mesenchymal stem cells; GAPDH, glyceraldehyde-3-phosphate dehydrogenase.

HFMSCs were strongly positive for MSC surface markers such as CD90, CD105, CD49d, and CD73 on flow cytometric analysis. Conversely, HFMSCs were negative for hematopoietic stem cell surface markers, including CD14, CD34, HLA-DR, and CD45 (Fig. 1A). Moreover, HFMSCs exhibit a unique fibroblast-like structure and demonstrate the ability to differentiate multiple times. Lipid droplets were noted in the cytoplasm during adipogenic differentiation, and calcium deposits marked osteogenic and chondrogenic differentiation, suggesting the likelihood of chondrosphere development within the cells. Differentiation was achieved by applying Oil Red O, Alizarin Red S, and Alcian blue staining in that order (Fig. 1B to D). These findings indicated that the isolated cells possessed typical HFMSC characteristics. Furthermore, we obtained cell culture supernatants from the HFMSCs and extracted the exosomes (Fig. 1E). As shown in Fig. 1F, transmission electron microscopy images revealed that HFMSC-Exos exhibited a cup or spherical shape. Furthermore, a NanoSight tracking analysis was conducted to ascertain the average exosome diameter (129 nm; Fig. 1G). A Western blotting analysis was performed to validate the presence of recognized exosome-positive markers (CD9, CD63, and CD81) and exosome-negative markers (calnexin) (Fig. 1H). The gathered information robustly suggested that the nanoparticles displayed traits that aligned with those of clearly identified exosomes.

### HFMSC-Exos enhance HDF growth and movement while reducing aging indicator β-galactosidase levels

**Fig. 2.**
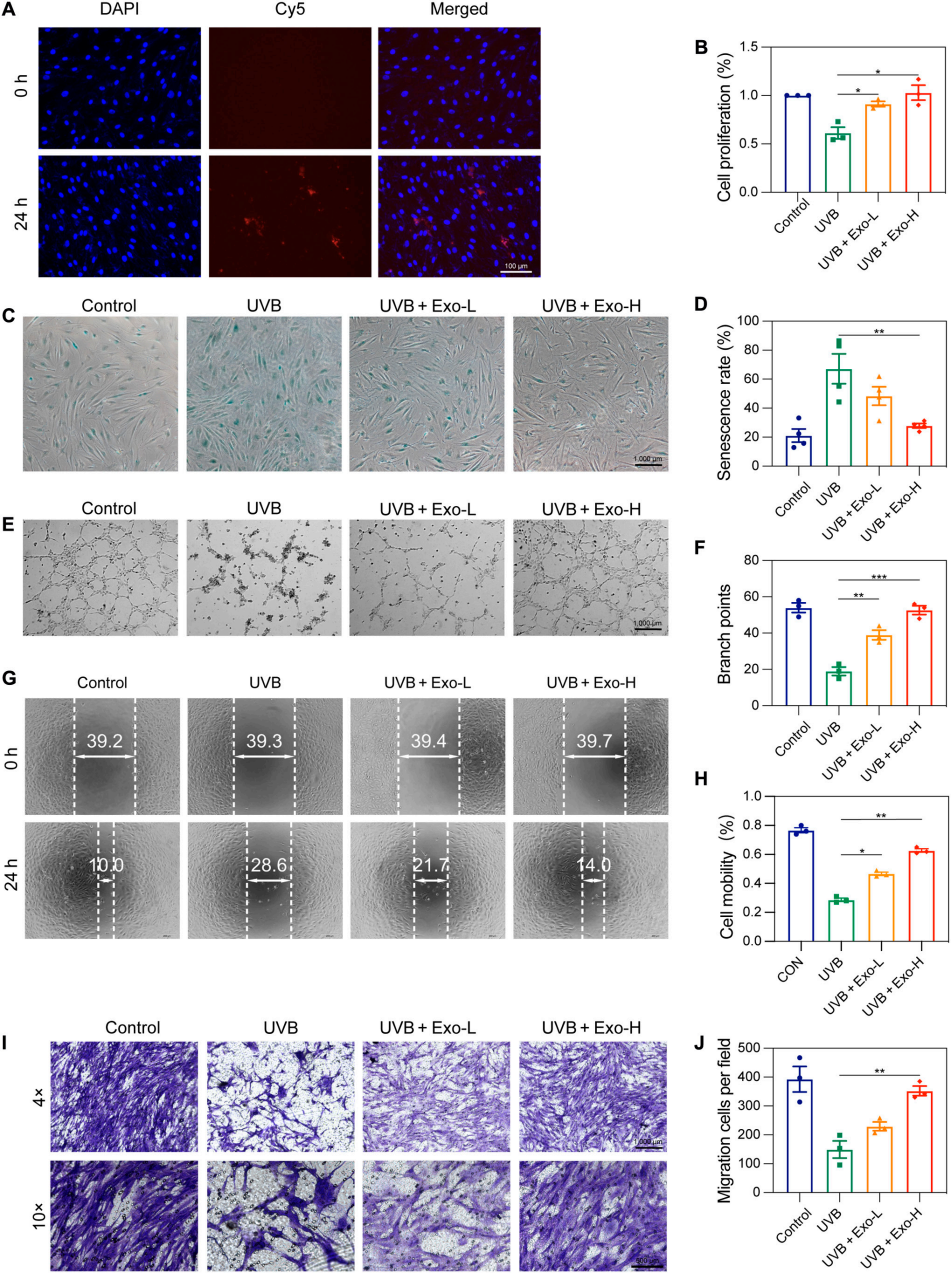
Impact of HFMSC-Exos on HDF proliferation and migration. (A) Representative image of DiR-labeled HFMSC-Exos internalized by HDFs (scale bar = 100 μm). (B) Analysis of HDF proliferation rate after exposure to HFMSC-Exos. (C) Representative image of HDF β-galactosidase expression after exposure to HFMSC-Exos (scale bar = 1,000 μm). (D) Proportion of senescent cells in each group in panel (C). (E and F) Representative images of the angiogenic capacity of human umbilical vein endothelial cells exposed to HFMSC-Exos analyzed by angiogenesis assay (scale bar = 1,000 μm). (G and H) The impact of HFMSC-Exos on HDF migration was evaluated using a scratch assay and a transwell migration assay (scale bar = 200 μm). (I and J) Statistical analyses were performed of the scratch migration rates of panels (G) and (H) and the number of cells crossed in the transwell assay (scale bar = 1,000 μm). Data are shown as mean ± standard error of the mean (SEM) (*P < 0.05; **P < 0.01). HDF, human dermal fibroblast; HFMSC, human hair follicle mesenchymal stem cells; HFMSC-Exos, exosomes derived from human hair follicle mesenchymal stem cells; MSCs, mesenchymal stem cells; DAPI, 4′,6-diamidino-2-phenylindole; UVB, ultraviolet B.

We hypothesized that exosomes, an essential element of HFMSC-conditioned medium, could be crucial to reducing skin photoaging. To evaluate the impact of HFMSC-Exos on UV-irradiated HDFs, we initially investigated the uptake of HFMSC-Exos by HDFs. The localization of DiR-labeled HFMSC-Exos was observed in the perinuclear and nuclear regions of HDFs (Fig. 2A). We determined the dosing concentration of HFMSC-Exos through the Cell Counting Kit-8 experiment, as shown in Fig. S1. Additionally, HFMSC-Exos have the potential to boost the growth and movement of UV-exposed HDFs following a 24-h stimulation phase. The observations indicated an increased growth rate of HDFs correlating with varying treatment scenarios (Fig. 2B). In the β-galactosidase assay, the senescence-associated β-galactosidase kit was employed to detect β-galactosidase expression in HDFs treated with HFMSC-Exos. HFMSC-Exos down-regulated β-galactosidase expression in HDFs, resulting in a marked decrease in the proportion of senescent cells (Fig. 2C and D). The cellular migration analysis demonstrated a marked enhancement in HDF migration with HFMSC-Exo stimulation. This was observed through the wound scratch and transwell migration analyses, with a statistically marked difference in the relative scratch area between the HFMSC-Exos and UVB-irradiated groups (*P* < 0.05) (Fig. 2G to J). Furthermore, HFMSC-Exos promoted HUVEC angiogenesis (Fig. 2E and F). We investigated the effect of HFMSC-Exo on the apoptosis and cell cycle of HDF cells. From Figs. S2 and S3, it can be seen that HFMSC-Exos have no substantial effect on the apoptosis and cell cycle of HDF cells.

### HFMSC-Exos attenuate photoaging by attenuating reactive oxygen species expression and upregulating HDF expression of COL-1/3

**Fig. 3.**
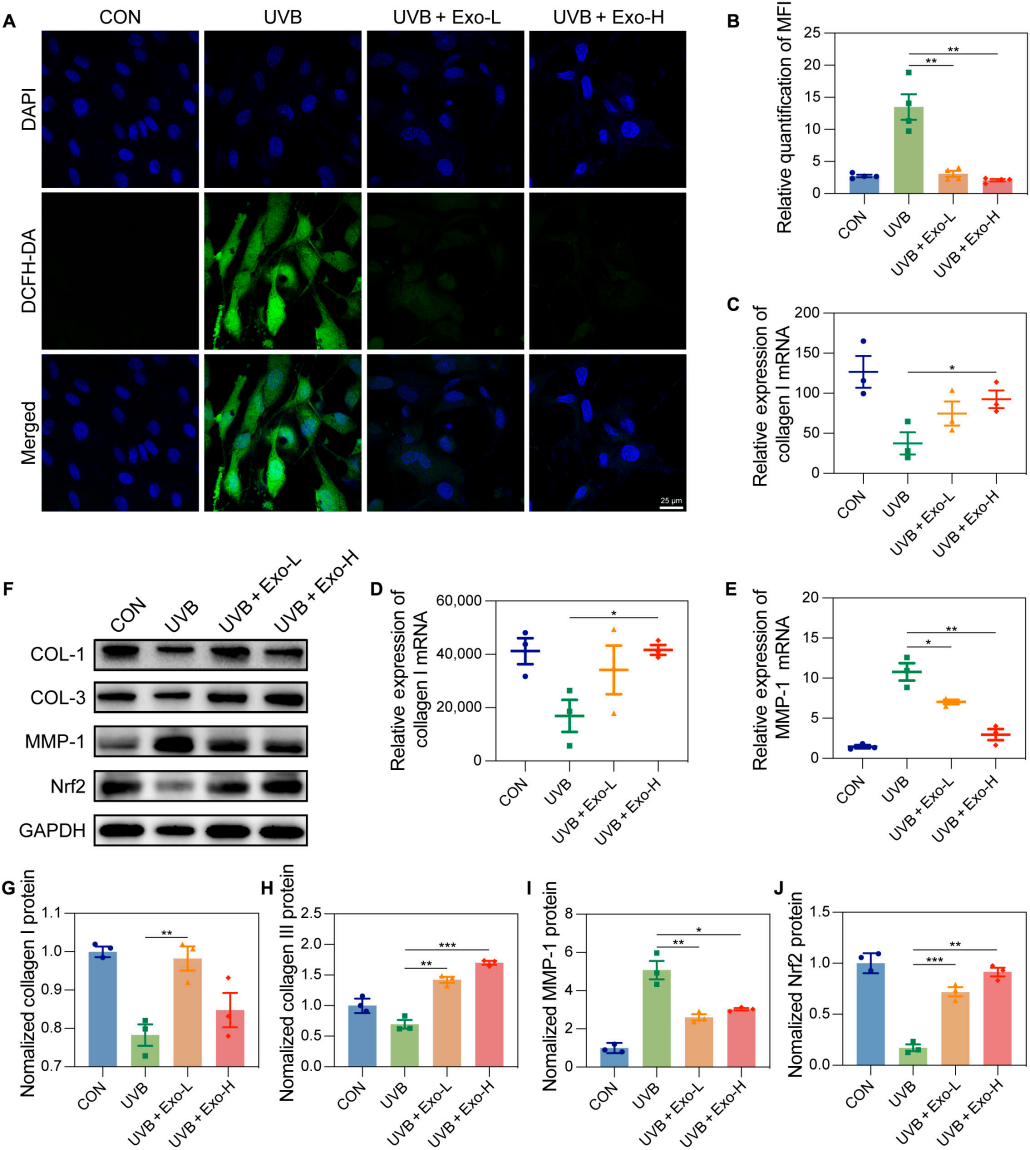
HFMSC-Exos accelerated extracellular matrix remodeling by inhibiting the generation of reactive oxygen species and upregulating collagen expression. (A) Representative images of dichlorodihydrofluorescein diacetate staining to detect the ability of HFMSC-Exos to scavenge intracellular reactive oxygen species from HDFs; scale bar = 25 μm. (B) Relative quantitative plots of fluorescence intensities for each group in panel (A). The fluorescence intensities of reactive oxygen species were markedly decreased in HDFs exposed to HFMSC-Exos compared to that of the ultraviolet B group, and this variance held statistical significance. (C to E) Quantitative real-time polymerase chain reaction plots of relative quantification of COL-1, COL-3, and MMP-1 messenger RNA (mRNA) expression in HDFs exposed to HFMSC-Exos, with statistically marked differences. (F) Bands of protein expression of COL-1, COL-3, MMP-1, and NF-E2-related factor 2 in HDFs exposed to HFMSC-Exos detected by Western blotting. (G to J) The relative quantitative statistics of COL-1, COL-3, MMP-1, and NF-E2-related factor 2 protein expression are plotted. Quantitative data were normalized against glyceraldehyde-3-phosphate dehydrogenase, and the differences were statistically marked. Data are shown as mean ± SEM (*P < 0.05; **P < 0.01; ***P < 0.001). HDFs, human dermal fibroblasts; HFMSCs, human hair follicle mesenchymal stem cells; HFMSC-Exos, exosomes derived from human hair follicle mesenchymal stem cells; COL-1, collagen type 1; COL-3, collagen type 3; MMP-1, matrix metalloproteinase 1; MSCs, mesenchymal stem cells; CON, control; DCFH-DA, dichlorodihydrofluorescein diacetate; Nrf2, NF-E2-related factor 2; MFI, mean fluorescence intensity.

HFMSC-Exos exhibited the ability to decrease the expression of reactive oxygen species in HDFs as evidenced by a marked decrease in the fluorescence intensity of DCFH-DA compared to that in the UV-irradiated group (Fig. 3A and B). Furthermore, HFMSC-Exos enhanced the mRNA levels of COL-1 and COL-3 and reduced the mRNA levels of MMP-1 in HDFs (Fig. 3C to E). These alterations were statistically marked (*P* < 0.05) compared with those of the UVB group, which demonstrated a similar trend at the protein level (Fig. 3F to I). NF-E2-related factor 2 enhances cellular protection against oxidative stress and damage. Our results indicated a marked increase in NF-E2-related factor 2 protein expression in HDFs exposed to HFMSC-Exos compared to that of the UVB group (Fig. 3F and J). These findings suggest that HFMSC-Exos promote the expression of COL-1/COL-3, which are major components of the extracellular matrix, leading to accelerated extracellular matrix remodeling. Moreover, HFMSC-Exos down-regulated MMP-1 expression and attenuated collagen fiber degradation.

### HFMSC-Exos reduce wrinkles and exert antiphotoaging effects in a photoaging nude mouse model

The main challenge in using animal models for skin photoaging research stems from natural differences in skin structure and subcutaneous tissue between humans and animals [21]. Among the various species employed to model photoaging, hairless nude mice are the most commonly used. Irradiation of the dorsal skin of nude mice with UV light is a widely accepted method for investigating cutaneous photoaging. This preference is attributed in part to the hairless nature of nude mice, which facilitates the observation of skin-specific alterations resulting from UV irradiation or subsequent treatments compared to other animal models. This study specifically focused on examining the structure and arrangement of collagen.

**Fig. 4.**
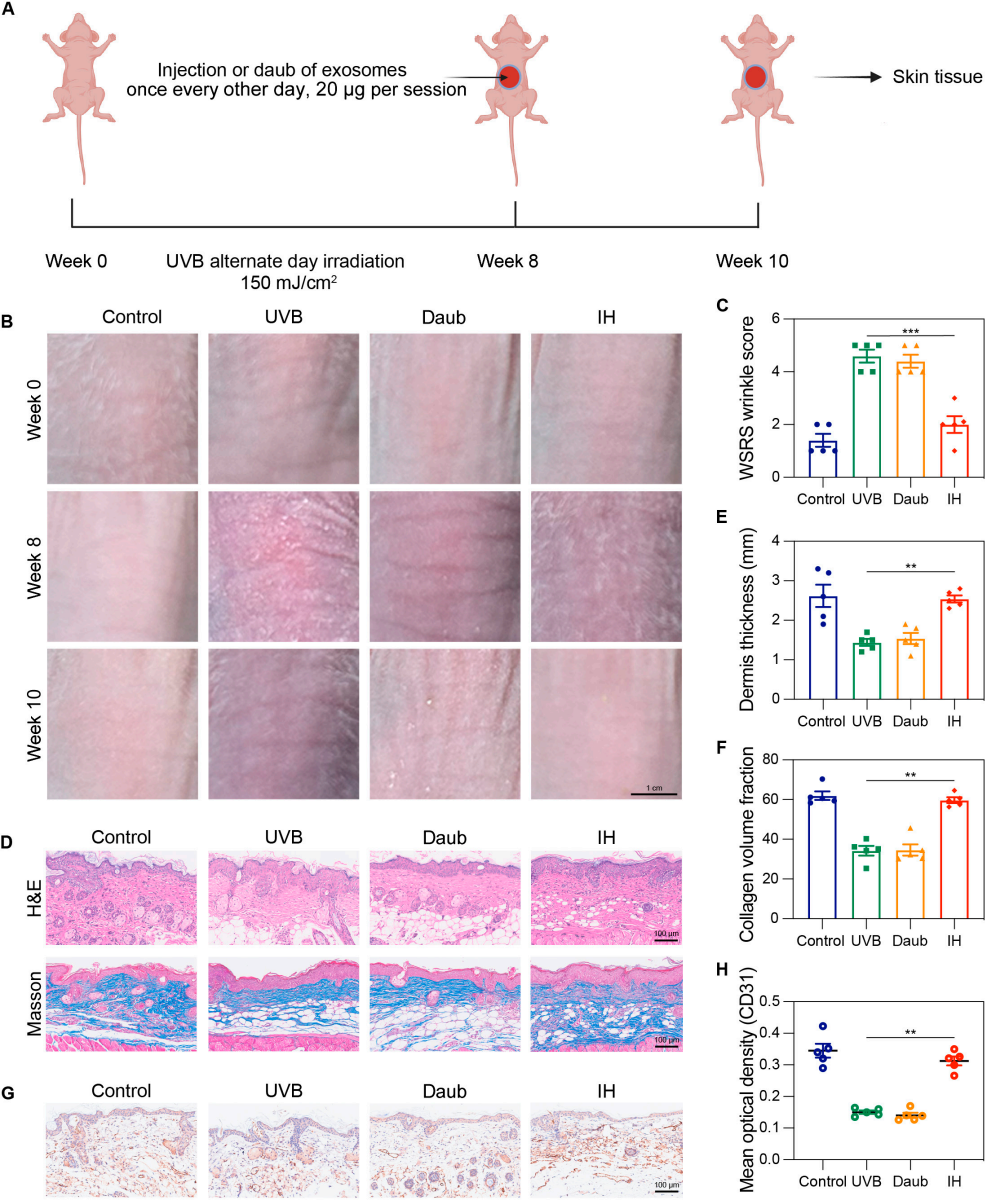
Effect of HFMSC-Exos on a photoaging model in nude mice. (A) Schematic diagram of the experimental procedure. (B) Digital photographs of the dorsal skin areas after ultraviolet B irradiation at weeks 0 and 8 and treatment with HFMSC-Exos. (C) Bar graphs showing the Wrinkle Severity Rating Scale scores of each group. (D) Representative images of H&E and Masson’s trichrome staining of dorsal skin after 2 weeks of treatment with HFMSC-Exos (scale bar = 100 μm). (E and F) Relative quantitative plots of dermal thickness and collagen fibers in sections. (G and H) Representative immunohistochemical images of CD31 in mouse skin tissue and graphs of the relative orientation of CD31. Data are shown as mean ± standard error (*P < 0.05; **P < 0.01; ***P < 0.001). H&E, hematoxylin and eosin; HFMSCs, human hair follicle mesenchymal stem cells; HFMSC-Exos, exosomes derived from human hair follicle mesenchymal stem cells; MMP-1, matrix metalloproteinase 1; MSCs, mesenchymal stem cells; WSRS, Wrinkle Severity Rating Scale; IH, immunohistochemical staining.

To investigate the impact of HFMSC-Exos in the animal model, the backs of nude mice were exposed to continuous UVB irradiation for 8 weeks to induce photoaging defects. Subsequently, HFMSC-Exos were administered by subcutaneous injection or daub (Fig. 4A). An analysis of digital photographs revealed that the subcutaneous injection of HFMSC-Exos markedly facilitated the recovery of skin wrinkles, whereas the topical application did not yield a noticeable effect, indicating inadequate skin permeability of HFMSC-Exos. These findings were obtained after 2 weeks of HFMSC-Exo treatment, and a statistically marked difference (*P* < 0.05) in skin wrinkles was observed after HFMSC-Exo treatment versus pretreatment (Fig. 4B and C). Furthermore, hematoxylin and eosin and Masson’s trichrome staining were used to assess the thickness and degree of collagen accumulation after dermal application (Fig. 4D to F). We also examined the angiogenic marker CD31 in the skin removed from the mice; as shown in Fig. 4G and H, the subcutaneous injection of exosomes promoted CD31 production. The results revealed a thicker dermis with increased collagen fiber production in the HFMSC-Exo subcutaneous injection treatment group. In vivo studies further confirmed the antiphotoaging effects of HFMSC-Exos and proposed possible treatment approaches for clinical use.

### HFMSC-Exos promote collagen production in the photoaging nude mouse model

**Fig. 5.**
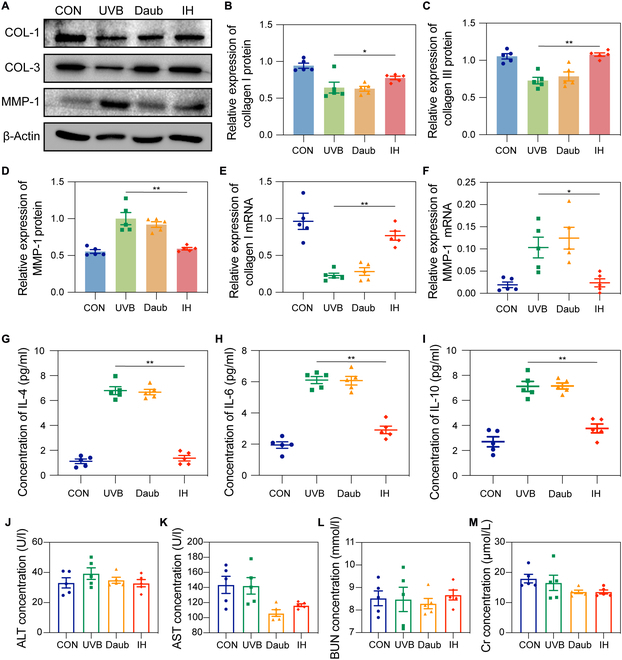
Antiphotoaging properties and safety assessment of HFMSC-Exos in vivo. (A) Representative Western blotting images of COL-1, COL-3, and MMP-1 in skin tissues. (B to D) Statistical analysis of histograms against the Western blotting images shown in panel (A). The differences were statistically marked. (E and F) Statistical histograms of the relative expressions of COL-1 and MMP-1 mRNA obtained by reverse transcription polymerase chain reaction; the differences were statistically marked. (G to I) Statistical representations of the levels of inflammatory agents interleukin-4, interleukin-6, and interleukin-10 in mice. (J to M) Biochemical analyses of the statistical images of ALT, AST, BUN, and Cr obtained from mice in vivo (*P < 0.05; **P < 0.01). ALT, alanine aminotransferase; AST, aspartate aminotransferase; BUN, blood urea nitrogen; Cr, creatinine; HFMSC-Exos, exosomes derived from human hair follicle mesenchymal stem cells; IH, immunohistochemical staining; IL-4, interleukin-4; IL-6, interleukin-6; IL-10, interleukin-10; MMP-1, matrix metalloproteinase 1.

To investigate the impact of HFMSC-Exos on collagen synthesis in the dermis of photoaged nude mice, skin tissues were obtained following 2-week treatment with HFMSC-Exos to evaluate the expression levels of collagen and MMP-1. Collected skin tissues were analyzed to determine the expression levels of COL-1, COL-3, and MMP-1. The results depicted in Fig. 5A to D demonstrate substantial upregulation of COL-1 and COL-3 expression as well as down-regulation of MMP-1 expression induced by HFMSC-Exo treatment. These findings indicated the promotion of dermal extracellular matrix remodeling, with a statistically marked difference observed between the 2 experimental groups (*P* < 0.05). The results presented in Fig. 5E and F demonstrated interpretive consistency of the aforementioned findings at the mRNA level.

We tested the sera obtained from mice for inflammatory factors such as interleukin-4, interleukin-6, and interleukin-10 and found that the concentrations in light-aged mice had increased markedly, suggesting that light aging causes inflammation in mice. When HFMSC-Exos were injected subcutaneously, all of the above inflammatory factors returned to normal levels. These results indicate that HFMSC-Exos can reduce inflammation in light-aged mice (Fig. 5G to I). To assess the safety of HFMSC-Exos in vivo, biochemical assays were conducted on sera obtained from the mice. As depicted in Fig. 5J to M, the levels of glutamic oxaloacetic aminotransferase, glutamic alanine aminotransferase, urea nitrogen, and creatinine clearance in the HFMSC-Exo-treated mice were comparable to those in the control group, suggesting favorable biocompatibility of HFMSC-Exos. We also conducted statistical analysis on the weight of mice during treatment to test the safety of HFMSC-Exos. As shown in Fig. S4, there was no substantial difference in mouse weight, indicating that HFMSC-Exos have good safety. These results reinforce the antiphotoaging effects of HFMSC-Exos and their viability as treatment methods in the clinical setting.

### HFMSC-Exos regulate the photoaging developmental process via upregulating relevant genes in the TGF-β1/Smad signaling pathway after HFMSC-Exo administration

**Fig. 6.**
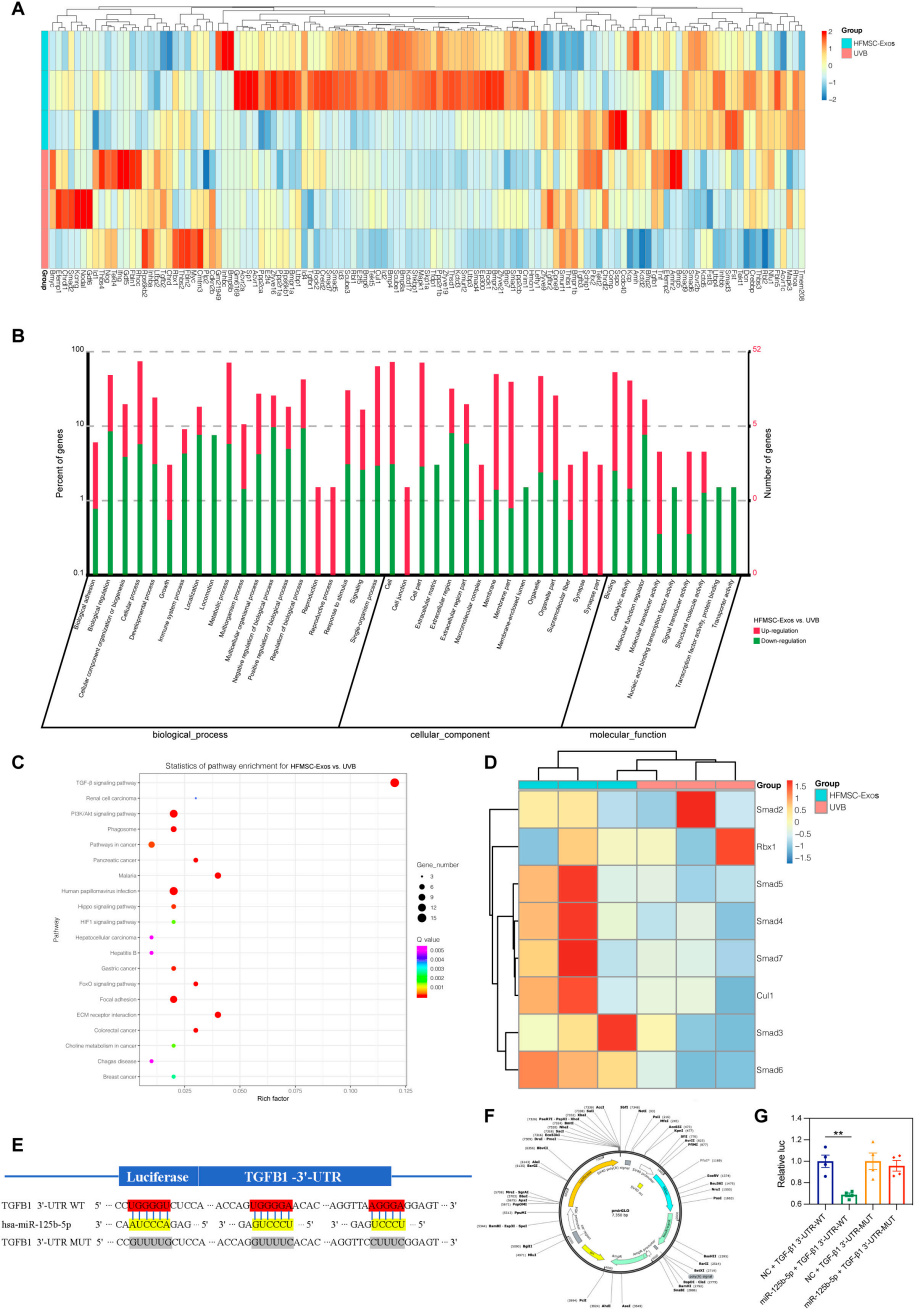
Analysis of RNA sequencing results. (A) Heatmap of differentially expressed genes of the TGF-β1/Smad pathway. (B) Gene Ontology plots for all differential genes. (C) Kyoto Encyclopedia of Genes and Genomes enrichment maps for all differential genes. (D) Heatmap of differentially expressed genes associated with the TGF-β1/Smad pathway Smad2/3. (E) Bioinformatics-predicted binding sites of miR-125b-5p to TGF-β1. (F and G) Reverse transcription polymerase chain reaction validation results of the dual-luciferase reporter (**P < 0.01). TGF-β1, transforming growth factor-β1; UTR, untranslated region; ECM, extracellular matrix; WT, wild type; MUT, mutant; luc, luciferase activity; NC, negative control.

We analyzed the RNA sequencing results of mouse skin tissues before and after dosing; as shown in Fig. 6A, a total of 123 genes in the TGF-β1/Smad pathway were differentially expressed after dosing. The Gene Ontology analysis plot shown in Fig. 6B shows that several genes related to tissue repair and extracellular matrix remodeling were upregulated after drug administration. In Fig. 6C, through Kyoto Encyclopedia of Genes and Genomes pathway analysis, a total of 123 genes were selected in the differential analysis and then intersected with the predicted target genes to take the intersection; finally, 25 genes were left, and we focused on the trend of the TGF-β1/Smad signaling pathway after drug administration. As shown in Fig. 6D, we performed a heatmap analysis of the changes that occurred in the 8 genes of interest for self-screening and observed that Smad2/3 and the others were upregulated. Finally, our analyses via bioinformatics algorithms (TargetScan 7.0, PicTar, and miRanda) showed that miR-125b-5p contains the corresponding pairing of the TGF-β1 3′-UTR target region positions 2012 to 2019 (Fig. 6E).

A luciferase reporter assay was performed to confirm the target relationship. The miR-125b-5p mimics markedly reduced the luciferase activity of HEK293 cells transfected with a reporter plasmid containing the TGF-β1 wild-type 3′-UTR sequence, with a statistically marked difference versus miR-125b-5p-mimicked negative controls (*P* < 0.001), whereas the mutant TGF-β1 3′-UTR controls did not appear to be inhibited, indicating no binding (Fig. 6F and G).

### MiR-125b-5p in HFMSC-Exos exerts antiphotoaging effects in HDFs by directly targeting TGF-β1 and affecting the Smad pathway

**Fig. 7.**
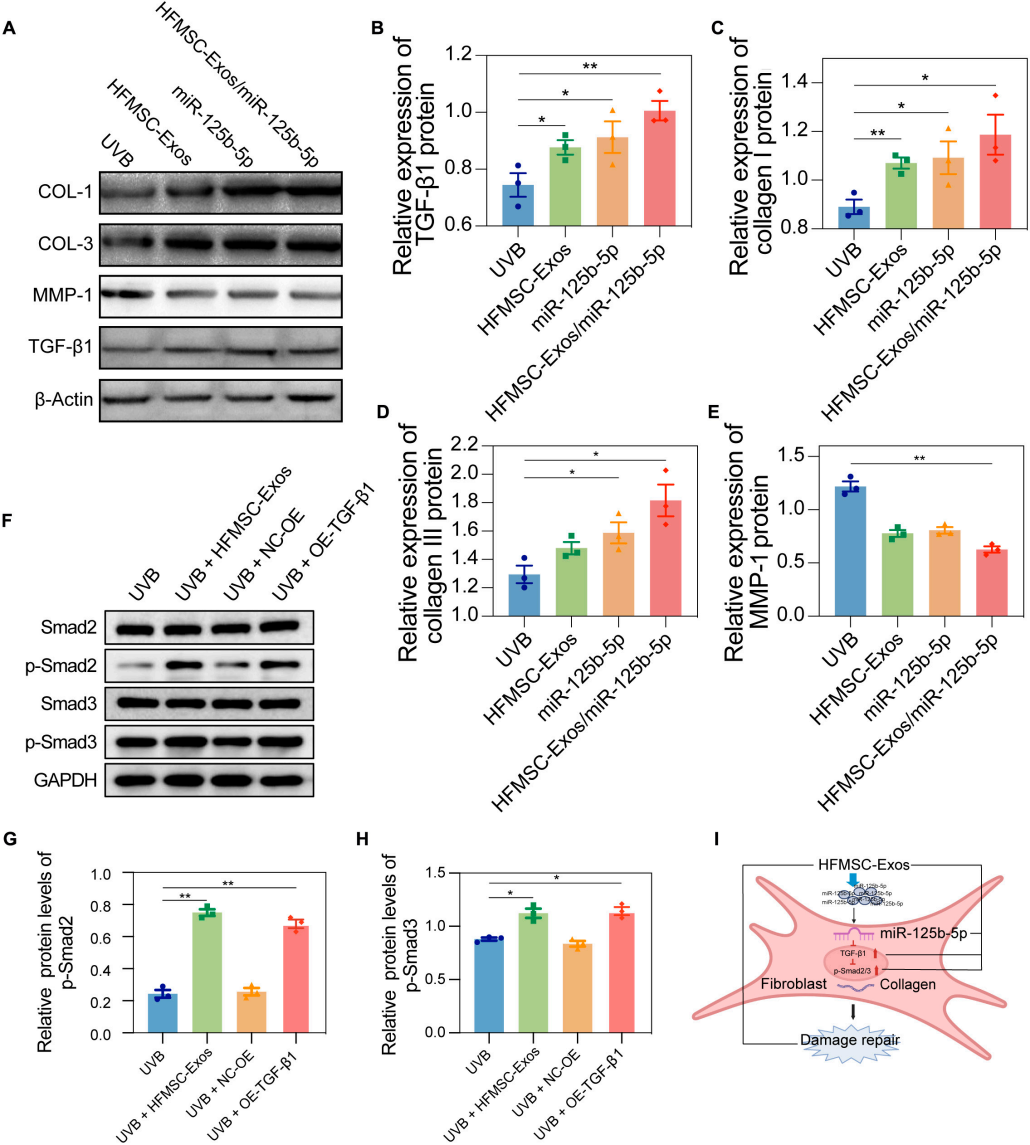
Validation of miRNA-125b-5p in relation to TGF-β1 targeting and its effect on the Smad pathway. (A) Representative Western blotting images of microRNA (miRNA) regulation of TGF-β1 and downstream COL-1, COL-3, and MMP-1. (B to E) Statistical images of the proteins shown in panel (A). (F) Representative effects of miRNA-125b-5p on Smad2/3 and phosphorylated protein Western blotting images. (G and H) Relative quantitative statistical images of phosphorylated proteins shown in panel (F). (I) Schematic representation of experimental results (*P < 0.05; **P < 0.01). COL-1, collagen type 1; COL-3, collagen type 3; MMP-1, matrix metalloprotein 1; TGF-β1, transforming growth factor-β1; OE, overexpression.

The ensuing investigation aimed to unravel the exact process behind the antiphotoaging properties of HFMSC-Exos. The results clearly indicate a substantial link between the antiphotoaging impact of exosome miR-125b-5p from HFMSC on HDFs and TGF-β1. Accordingly, we aimed to investigate the regulatory interplay between miR-125b-5p and TGF-β1 in managing photoaging. HDFs were transfected with miR-125b-5p mimics or inhibitors accompanied by their respective negative controls utilizing Lipofectamine 3000 reagent. The expressions of miR-125b-5p and TGF-β1 were evaluated by Western blot analysis. Notably, TGF-β1 expression was upregulated in HDFs transfected with the miR-125b-5p mimic (Fig. 7A to E and Fig. S5). These findings strongly suggest that TGF-β1 is a direct target of miR-125b-5p in the context of photoaging.

Given the well-established association between the Smad signaling pathway and photoaging diseases, our future investigations will focus on elucidating the effects of HFMSC-Exos and TGF-β1 overexpression on the Smad pathway. The results of this study indicated that HFMSC-Exos and TGF-β1 overexpression increase p-Smad2 or p-Smad-3 expression (Fig. 7F). This suggests that HFMSC-Exos or TGF-β1 overexpression can regulate Smad signaling to improve photoaging. A notable intergroup statistical disparity (*P* < 0.05) was observed (Fig. 7G and H). These results further clarify how HFMSC-Exos counteract photoaging via the miR-125b-5p/TGF-β1/Smad pathway (Fig. 7I).

## Discussion

This study presents the following primary findings: First, consistent with previous studies indicating the ability of stem cell-derived exosomes to enhance fibroblast proliferation, migration, and collagen synthesis [22], we confirmed that HFMSC-Exos facilitate the bioactivation and collagen deposition of HDFs, thereby exerting an antiphotoaging effect. These observed disparities can largely be attributed to specific fibroblast types, namely, normal and UV-irradiated HDFs, with our focus on the impact of HFMSC-Exos on UV-irradiated HDFs. Second, our study successfully demonstrated the potential of miR-125b-5p in HFMSC-Exos to mitigate the effects of photoaging on HDFs. Furthermore, we identified TGF-β1 as a direct target of miR-125b-5p and observed that it regulates the Smad pathway, ultimately promoting profibronectin expression in HDFs. Collectively, these findings elucidate the mechanism of the antiphotoaging properties of HFMSC-Exos through the miR-125b-5p/TGF-β1/Smad axis. This study provides substantial evidence supporting the therapeutic potential of HFMSC-Exos.

Photoaging is a chronic skin-damaging disease that poses an urgent clinical challenge. UV-exposed skin fibroblasts experience slower proliferation and increased MMP-1 levels, leading to extracellular matrix degradation and decreased collagen production [23,24]. Therefore, we assessed photoaging by measuring the expressions of COL-1, COL-3, and MMP-1.

Stem cells play an important role in mitigating skin diseases [25,26]. Previous studies reported that stem cell-conditioned medium has an antiphotoaging function [27]. Compared with other sources of stem cells, hair follicle-derived MSCs have shown substantial advantages in safety, source richness, age limitation, proliferation and differentiation ability, and sampling methods. These advantages make hair follicle-derived MSCs have broad application prospects and huge development potential in the field of stem cell therapy. Exosomes are 30 to 150 nm long and secreted by virtually all cell types into small extracellular vesicles in the culture supernatant [28,29]. Exosomes may be a promising therapeutic strategy because their receptors are less immunogenic and are noncytotoxic and nonmutagenic compared with other gene delivery vectors [30,31]. MSC-derived exosomes also promote wound healing [32]. Moreover, exosomes have emerged as important factors in the therapeutic progression of hepatic and lung injuries [33]. Accordingly, we demonstrated the antiphotoaging properties of HFMSC-Exos. Specifically, DiR-labeled HFMSC-Exos were internalized by HDFs and promoted their proliferation and migration. Additionally, HFMSC-Exos promoted collagen production in HDFs. More importantly, in a photoaging model of nude mice, HFMSC-Exos promoted the recovery of wrinkles and collagen deposition to inhibit photoaging. As described above, HFMSC-Exos inhibited photoaging both in vitro and in vivo. However, the mechanisms underlying these effects have not been completely elucidated.

Emerging evidence suggests that exosomes, that is, paracrine signaling mediators, influence the progression of photoaging diseases by transferring anti- or prophotoaging miRNAs to target cells, affecting pathological proteogenesis [34]. One of the major challenges in synthesizing miRNAs is their rapid degradation due to the high activity of plasma ribonucleases; however, exosomes would protect miRNAs from such degradation [35,36]. Our study reported that miR-125b-5p was highly expressed in HFMSC-Exos, whereas HFMSC-Exo-miR-125b-5p promoted collagen expression in HDFs. Collagen expression was promoted with miR-125b-5p mimics (HFMSC-Exo-miR-125b-5p) to emphasize the importance of miR-125b-5p in HFMSC-Exos.

Strikingly, the expressions of COL-1 and COL-3 increased and the expression of MMP-1 decreased in HDFs transfected with miR-125b-5p mimics. These results suggest that miR-125b-5p directly targets TGF-β1 to regulate photoaging. Therefore, we investigated the effects of TGF-β1 on photoaging in subsequent experiments.

TGF-β1 is ubiquitously expressed in many cell types in the human body and highly expressed in the bone marrow, thymus, and spleen [37]. In the skin, TGF-β1 is expressed mainly by fibroblasts, epithelial cells, smooth muscle cells, and microvascular endothelial cells [38,39]. However, its effects on skin fibroblasts have not yet been elucidated. This study explored the expression of TGF-β1 in photoaging and found that it was low in UV-irradiated HDF and its overexpression promoted the expression of collagen and down-regulated the expression of MMP-1 in HDFs. As described above, TGF-β1 overexpression promoted collagen deposition and extracellular matrix remodeling. Next, we explored the possible mechanisms underlying the effects of TGF-β1 on photoaging.

Photoaging is reportedly closely related to the Smad signaling pathway, and the Smad proteins are involved in signal transduction and transcriptional regulation [40,41]. In the canonical Smad pathway, phosphorylated Smad2 and Smad3 bind to Smad4 to form an Smad complex that translocates to the nucleus to regulate target gene transcription. A recent study showed that human umbilical cord MSC-Exos (hucMSC-Exos) significantly increased the expressions of collagen and p-Smad2 to alleviate skin damage [42]. In addition, MSC-Exos regulated the dermal repair of injured skin by upregulating the Smad signaling pathway, indicating that Smad2 phosphorylation gradually increased with increasing exosome concentration in the MSC and Exo treatment groups [43]. Moreover, the collagen and phosphorylation levels of Smad2/3 were significantly increased in fibroblasts treated with hucMSC-Exos, suggesting that hucMSC-Exos facilitate the dermal fibroblast–myofibroblast transition by promoting SMAD signaling pathway function [44]. Consistent with these findings, we observed elevated protein levels of p-Smad2 and p-Smad3 in HFMSC-Exo-stimulated HDFs.

In contrast, some researchers reported that adipose MSC-Exos effectively down-regulate the protein expression of p-Smad2/3 to attenuate tissue fibrosis [45]. This difference is attributed to the state of quiescent or activated fibroblasts. TGF-β1 is a key mediator of tissue remodeling and collagen deposition during photoaging [46]. TGF-β1 is known to phosphorylate Smad proteins such as Smad2 and Smad3 [47,48]. Here, we observed upregulated expressions of p-Smad2 and p-Smad3. As mentioned above, these findings suggest that HFMSC-Exo or TGF-β1 overexpression modulates the Smad signaling pathway to exert antiphotoaging effects in HDF. There is evidence that the miR-125b-5p promoter contains a sequence complementary to Smad [49], which may be another viable mechanism associated with photoaging. Thus, we draw the preliminary conclusion that HFMSC-Exos attenuate photoaging through the miR-125b-5p/TGF-β1/Smad axis. In this study, HFMSC-Exos effectively promoted HDF bioactivity, collagen deposition, wrinkle recovery, and collagen synthesis in a photoaging nude mouse model. Furthermore, we verified that miR-125b-5p in HFMSC-Exos could directly target TGF-β1 to regulate the Smad pathway in photoaging. Our study provides a new therapeutic strategy and elucidates the specific mechanisms underlying the clinical treatment of photoaging.

## Ethical Approval

The collection and use of hair follicle MSCs were approved by Zhejiang Provincial People’s Hospital (ZN-20221214-0200-01). Relevant studies were conducted at the Experimental Animal Centre of Zhejiang Provincial People’s Hospital (20230606162017188974).

## Data Availability

All data of this study are available from the corresponding authors upon reasonable request.

## References

[1] Stratigos AJ, Katsambas AD. The role of topical retinoids in the treatment of photoaging. Drugs. 2005;65(8):1061–1072. 15907143 10.2165/00003495-200565080-00003

[2] Ichihashi M, Ando H. The maximal cumulative solar UVB dose allowed to maintain healthy and young skin and prevent premature photoaging. Exp Dermatol. 2014;23(Suppl 1):43–46. 25234836 10.1111/exd.12393

[3] Battie C, Jitsukawa S, Bernerd F, Del Bino S, Marionnet C, Verschoore M. New insights in photoaging, UVA induced damage and skin types. Exp Dermatol. 2014;23(Suppl 1):7–12. 10.1111/exd.12388 25234829

[4] Bian D, Wu Y, Song G, Azizi R, Zamani A. The application of mesenchymal stromal cells (MSCs) and their derivative exosome in skin wound healing: A comprehensive review. Stem Cell Res Ther. 2022;13(1): Article 24. 10.1186/s13287-021-02697-9 PMC8785459 35073970

[5] Botchkarev VA, Kishimoto J. Molecular control of epithelial–mesenchymal interactions during hair follicle cycling. J Investig Dermatol Symp Proc. 2003;8(1):46–55. 10.1046/j.1523-1747.2003.12171.x 12894994

[6] Cha H, Hong S, Park JH, Park HH. Stem cell-derived exosomes and nanovesicles: Promotion of cell proliferation, migration, and anti-senescence for treatment of wound damage and skin ageing. Pharmaceutics. 2020;12(12): Article 1135. 33255430 10.3390/pharmaceutics12121135 PMC7761250

[7] Chen S, He Z, Xu J. Application of adipose-derived stem cells in photoaging: Basic science and literature review. Stem Cell Res Ther. 2020;11(1): Article 491. 33225962 10.1186/s13287-020-01994-z PMC7682102

[8] Croston TL, Lemons AR, Beezhold DH, Green BJ. MicroRNA regulation of host immune responses following fungal exposure. Front Immunol. 2018;9: Article 170. 29467760 10.3389/fimmu.2018.00170 PMC5808297

[9] Ho PTB, Clark IM, Le LTT. MicroRNA-based diagnosis and therapy. Int J Mol Sci. 2022;23(13): Article 7167. 35806173 10.3390/ijms23137167 PMC9266664

[10] Gao W, Yuan LM, Zhang Y, Huang FZ, Gao F, Li J, Xu F, Wang H, Wang YS. miR-1246-overexpressing exosomes suppress UVB-induced photoaging via regulation of TGF-β/Smad and attenuation of MAPK/AP-1 pathway. Photochem Photobiol Sci. 2023;22(1):135–146. 36114328 10.1007/s43630-022-00304-1

[11] Yan T, Huang L, Yan Y, Zhong Y, Xie H, Wang X. Bone marrow mesenchymal stem cell-derived exosome miR-29b-3p alleviates UV irradiation-induced photoaging in skin fibroblast. Photodermatol Photoimmunol Photomed. 2023;39(3):235–245. 35950642 10.1111/phpp.12827

[12] Zhang Y, Liao JM, Zeng SX, Lu H. p53 downregulates Down syndrome-associated DYRK1A through miR-1246. EMBO Rep. 2011;12(8):811–817. 21637297 10.1038/embor.2011.98 PMC3147276

[13] Liao JM, Zhou X, Zhang Y, Lu H. MiR-1246: A new link of the p53 family with cancer and Down syndrome. Cell Cycle. 2012;11(14):2624–2630. 22751441 10.4161/cc.20809 PMC3409007

[14] Jennings JC, Mohan S, Linkhart TA, Widstrom R, Baylink DJ. Comparison of the biological actions of TGF beta-1 and TGF beta-2: Differential activity in endothelial cells. J Cell Physiol. 1988;137(1):167–172. 3170656 10.1002/jcp.1041370120

[15] Lin W, Xiong J, Jiang Y, Liu H, Bian J, Wang J, Shao Y, Ni B. Fibrillin-1 mutation contributes to Marfan syndrome by inhibiting Cav1.2-mediated cell proliferation in vascular smooth muscle cells. Channels (Austin). 2023;17(1): Article 2192377. 36972239 10.1080/19336950.2023.2192377 PMC10054150

[16] Xiong Y, Huang X, Jiao Y, Zhou C, Yu T. Synergistic effect of Mn-Si-COS on wound immune microenvironment by inhibiting excessive skin fibrosis mediated with ROS/TGF-β1/Smad7 signal. Biomater Adv. 2023;152: Article 213497. 37321008 10.1016/j.bioadv.2023.213497

[17] Park HN, Song MJ, Choi YE, Lee DH, Chung JH, Lee ST. LRG1 promotes ECM integrity by activating the TGF-β signaling pathway in fibroblasts. Int J Mol Sci. 2023;24(15): Article 12445. 37569820 10.3390/ijms241512445 PMC10418909

[18] Takano K, Kasamatsu S, Aoki M, Takahashi Y. Carbon dioxide-induced decrease in extracellular pH enhances the production of extracellular matrix components by upregulating TGF-β1 expression via CREB activation in human dermal fibroblasts. Exp Dermatol. 2023;32(10):1651–1622. 37377319 10.1111/exd.14867

[19] Zhou X, Ye H, Wang X, Sun J, Tu J, Lv J. Ursolic acid inhibits human dermal fibroblasts hyperproliferation, migration, and collagen deposition induced by TGF-β via regulating the Smad2/3 pathway. Gene. 2023;867: Article 147367. 36931410 10.1016/j.gene.2023.147367

[20] Théry C, Amigorena S, Raposo G, Clayton A. Isolation and characterization of exosomes from cell culture supernatants and biological fluids. Curr Protoc Cell Biol. 2006;30(1):3.22.1–3.22.29. 10.1002/0471143030.cb0322s30. 18228490

[21] Fan Y, Jeong JH, You GY, Park JU, Choi TH, Kim S. An experimental model design for photoaging. J Craniofac Surg. 2015;26(6):e467–e471. 26267568 10.1097/SCS.0000000000001902

[22] Zhang W, Bai X, Zhao B, Li Y, Zhang Y, Li Z, Wang X, Luo L, Han F, Zhang J, et al. Cell-free therapy based on adipose tissue stem cell-derived exosomes promotes wound healing via the PI3K/Akt signaling pathway. Exp Cell Res. 2018;370(2):333–342. 29964051 10.1016/j.yexcr.2018.06.035

[23] Kligman LH. Photoaging. Manifestations, prevention, and treatment. Clin Geriatr Med. 1989;5(1):235–251. 2646001

[24] Flament F, Saint-Leger D. Photoaging’s portrait: The road map towards its photoprotection. Int J Cosmet Sci. 2023;45(Suppl 1):33–44. 37638664 10.1111/ics.12903

[25] Deng M, Yu TZ, Li D, Wang X, Zhou G, Liu W, Cao Y, Xia W, Li W, Zhang WJ. Human umbilical cord mesenchymal stem cell-derived and dermal fibroblast-derived extracellular vesicles protect dermal fibroblasts from ultraviolet radiation-induced photoaging in vitro. Photochem Photobiol Sci. 2020;19(3):406–414. 32125331 10.1039/c9pp00421a

[26] Zou X, Zou D, Li L, Yu R, Li X, Du X, Guo J, Wang K, Liu W. Multi-omics analysis of an in vitro photoaging model and protective effect of umbilical cord mesenchymal stem cell-conditioned medium. Stem Cell Res Ther. 2022;13(1): Article 435. 36056394 10.1186/s13287-022-03137-y PMC9438153

[27] Liu Q, Luo Z, He S, Peng X, Xiong S, Wang Y, Zhong X, Zhou X, Eisenberg CA, Gao BZ. Conditioned serum-free medium from umbilical cord mesenchymal stem cells has anti-photoaging properties. Biotechnol Lett. 2013;35(10):1707–1714. 23690049 10.1007/s10529-013-1242-2

[28] Doyle LM, Wang MZ. Overview of extracellular vesicles, their origin, composition, purpose, and methods for exosome isolation and analysis. Cells. 2019;8(7): Article 727. 31311206 10.3390/cells8070727 PMC6678302

[29] He C, Zheng S, Luo Y, Wang B. Exosome theranostics: Biology and translational medicine. Theranostics. 2018;8(1):237–255. 29290805 10.7150/thno.21945 PMC5743472

[30] Kalluri R, LeBleu VS. The biology, function, and biomedical applications of exosomes. Science. 2020;367(6478): Article eaau6977. 32029601 10.1126/science.aau6977 PMC7717626

[31] Lai JJ, Chau ZL, Chen SY, Hill JJ, Korpany KV, Liang NW, Lin LH, Lin YH, Liu JK, Liu YC, et al. Exosome processing and characterization approaches for research and technology development. Adv Sci. 2022;9(15): Article e2103222. 10.1002/advs.202103222 PMC9130923 35332686

[32] Zhang K, Yu L, Li FR, Li X, Wang Z, Zou X, Zhang C, Lv K, Zhou B, Mitragotri S, et al. Topical application of exosomes derived from human umbilical cord mesenchymal stem cells in combination with sponge spicules for treatment of photoaging. Int J Nanomedicine. 2020;15:2859–2872. 32368058 10.2147/IJN.S249751 PMC7185618

[33] Tan Y, Huang Y, Mei R, Mao F, Yang D, Liu J, Xu W, Qian H, Yan Y. HucMSC-derived exosomes delivered BECN1 induces ferroptosis of hepatic stellate cells via regulating the xCT/GPX4 axis. Cell Death Dis. 2022;13(4): Article 319. 35395830 10.1038/s41419-022-04764-2 PMC8993870

[34] Gao W, Zhang Y, Yuan L, Huang F, Wang YS. Long non-coding RNA H19-overexpressing exosomes ameliorate UVB-induced photoaging by upregulating SIRT1 via sponging miR-138. Photochem Photobiol. 2023;99(6):1456–1467. 36916469 10.1111/php.13801

[35] Liu J, Fan L, Yu H, Zhang J, He Y, Feng D, Wang F, Li X, Liu Q, Li Y, et al. Endoplasmic reticulum stress causes liver cancer cells to release exosomal miR-23a-3p and up-regulate programmed death ligand 1 expression in macrophages. Hepatology. 2019;70(1):241–258. 30854665 10.1002/hep.30607 PMC6597282

[36] Lv LL, Feng Y, Wu M, Wang B, Li ZL, Zhong X, Wu WJ, Chen J, Ni HF, Tang TT, et al. Exosomal miRNA-19b-3p of tubular epithelial cells promotes M1 macrophage activation in kidney injury. Cell Death Differ. 2020;27(1):210–226. 31097789 10.1038/s41418-019-0349-y PMC7206053

[37] Kim KK, Sheppard D, Chapman HA. TGF-β1 signaling and tissue fibrosis. Cold Spring Harb Perspect Biol. 2018;10(4): Article a022293. 28432134 10.1101/cshperspect.a022293 PMC5880172

[38] Baik JE, Park HJ, Kataru RP, Savetsky IL, Ly CL, Shin J, Encarnacion EM, Cavali MR, Klang MG, Riedel E, et al. TGF-β1 mediates pathologic changes of secondary lymphedema by promoting fibrosis and inflammation. Clin Transl Med. 2022;12(6): Article e758. 35652284 10.1002/ctm2.758 PMC9160979

[39] Li X, Zhai Y, Xi B, Ma W, Zhang J, Ma X, Miao Y, Zhao Y, Ning W, Zhou H, et al. Pinocembrin ameliorates skin fibrosis via inhibiting TGF-β1 signaling pathway. Biomol Ther. 2021;11(8): Article 1240. 10.3390/biom11081240 PMC8393190 34439906

[40] Choi SI, Han HS, Kim JM, Park G, Jang YP, Shin YK, Ahn HS, Lee SH, Lee KT. *Eisenia bicyclis* extract repairs UVB-induced skin photoaging in vitro and in vivo: Photoprotective effects. Mar Drugs. 2021;19(12): Article 693. 34940692 10.3390/md19120693 PMC8709268

[41] Jiang H, Zhou X, Chen L. Asiaticoside delays senescence and attenuate generation of ROS in UV-exposure cells through regulates TGF-β1/Smad pathway. Exp Ther Med. 2022;24(5): Article 667. 36237596 10.3892/etm.2022.11603 PMC9500490

[42] Wu P, Zhang B, Han X, Sun Y, Sun Z, Li L, Zhou X, Jin Q, Fu P, Xu W, et al. HucMSC exosome-delivered 14-3-3ζ alleviates ultraviolet radiation-induced photodamage via SIRT1 pathway modulation. Aging. 2021;13(8):11542–11563. 33882455 10.18632/aging.202851 PMC8109102

[43] Jiang T, Wang Z, Sun J. Human bone marrow mesenchymal stem cell-derived exosomes stimulate cutaneous wound healing mediates through TGF-β/Smad signaling pathway. Stem Cell Res Ther. 2020;11(1): Article 198. 32448395 10.1186/s13287-020-01723-6 PMC7245763

[44] Hu J, Chen Y, Huang Y, Su Y. Human umbilical cord mesenchymal stem cell-derived exosomes suppress dermal fibroblasts-myofibroblats transition via inhibiting the TGF-β1/Smad 2/3 signaling pathway. Exp Mol Pathol. 2020;115: Article 104468. 32445750 10.1016/j.yexmp.2020.104468

[45] Fang S, Xu C, Zhang Y, Xue C, Yang C, Bi H, Qian X, Wu M, Ji K, Zhao Y, et al. Umbilical cord-derived mesenchymal stem cell-derived exosomal microRNAs suppress myofibroblast differentiation by inhibiting the transforming growth factor-β/SMAD2 pathway during wound healing. Stem Cells Transl Med. 2016;5(10):1425–1439. 27388239 10.5966/sctm.2015-0367 PMC5031180

[46] Moon NR, Kang S, Park S. Consumption of ellagic acid and dihydromyricetin synergistically protects against UV-B induced photoaging, possibly by activating both TGF-β1 and wnt signaling pathways. J Photochem Photobiol B. 2018;178:92–100. 29128706 10.1016/j.jphotobiol.2017.11.004

[47] Lv Q, Wang J, Xu C, Huang X, Ruan Z, Dai Y. Pirfenidone alleviates pulmonary fibrosis in vitro and in vivo through regulating Wnt/GSK-3β/β-catenin and TGF-β1/Smad2/3 signaling pathways. Mol Med. 2020;26(1): Article 49. 32448163 10.1186/s10020-020-00173-3 PMC7245944

[48] Liu Z, Wang W, Li X, Tang S, Meng D, Xia W, Wang H, Wu Y, Zhou X, Zhang J. Capsaicin ameliorates renal fibrosis by inhibiting TGF-β1-Smad2/3 signaling. Phytomedicine. 2022;100: Article 154067. 35349832 10.1016/j.phymed.2022.154067

[49] Shen Z, Letra A, Silva RM. MicroRNAs markedly expressed in apical periodontitis cooperatively regulate cytokines and growth factors promoting an anti-inflammatory response. J Endod. 2023;49(3):286–293. 36627081 10.1016/j.joen.2022.12.013

