# Supplementary material for: Human Hair Follicle Mesenchymal Stem Cell-Derived Exosomes Attenuate UVB-Induced Photoaging via the miR-125b-5p/TGF-β1/Smad Axis
Source: Biomater Res. 2025 Jan 13;29:0121. doi: 10.34133/bmr.0121 (PMC11725759; doi:10.34133/bmr.0121)
Supplement: Supplementary 1 — Figs. S1 to S5 [file bmr.0121.f1.docx]

**Supplementary materials**

**
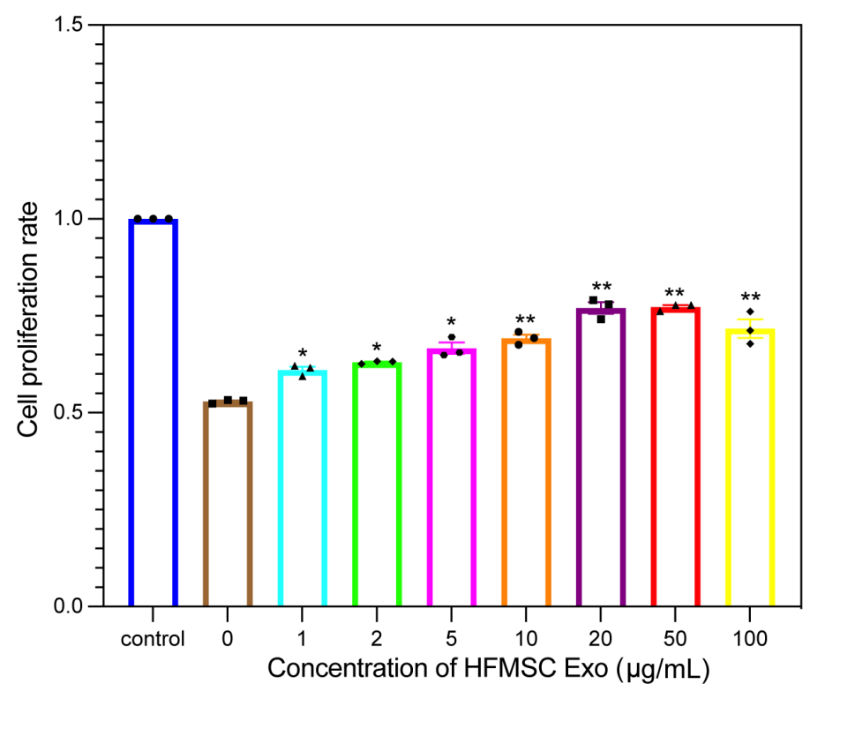
**

**Fig S1.** The effect of different concentrations of HFMSC Exo on promoting HDF cell proliferation. *p<0.05，**p<0.01.

**
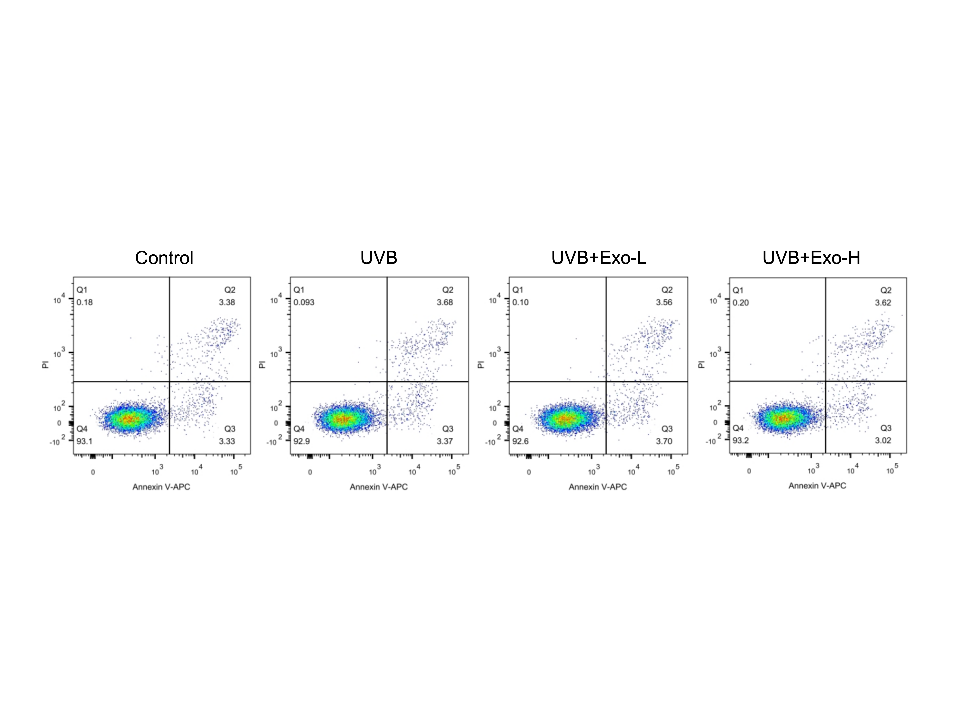
**

A

**
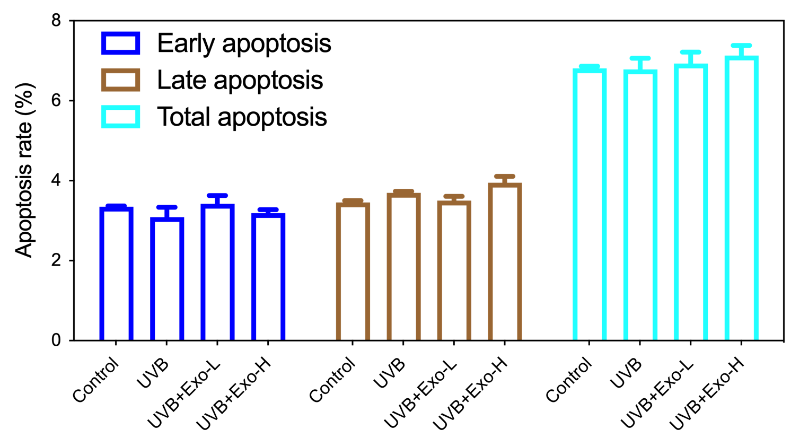
**

B

**Fig S2.** HFMSC-Exo has no effect on apoptosis of HDF cells. (A) Representative flow cytometry of the effect of HFMSC-Exo on apoptosis of HDF cells. (B) Apoptosis statistics of Figure A.

**
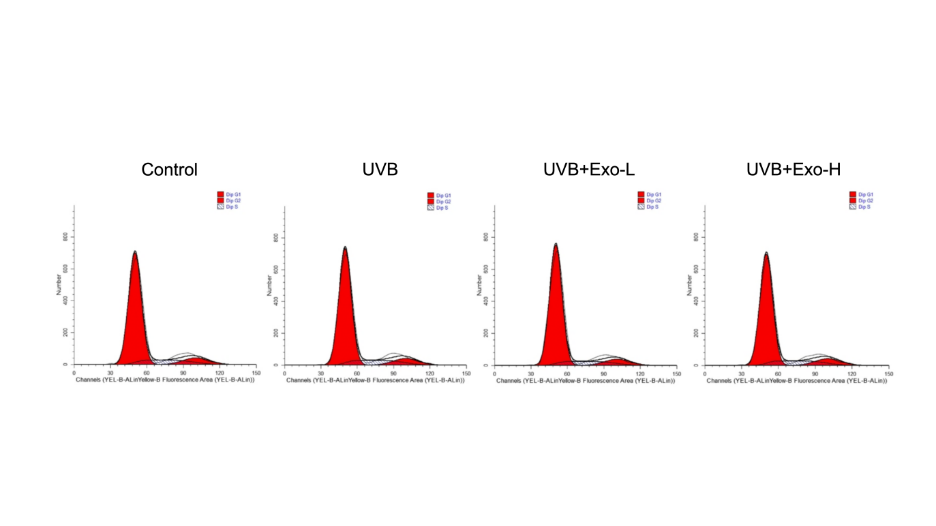
**

A

B


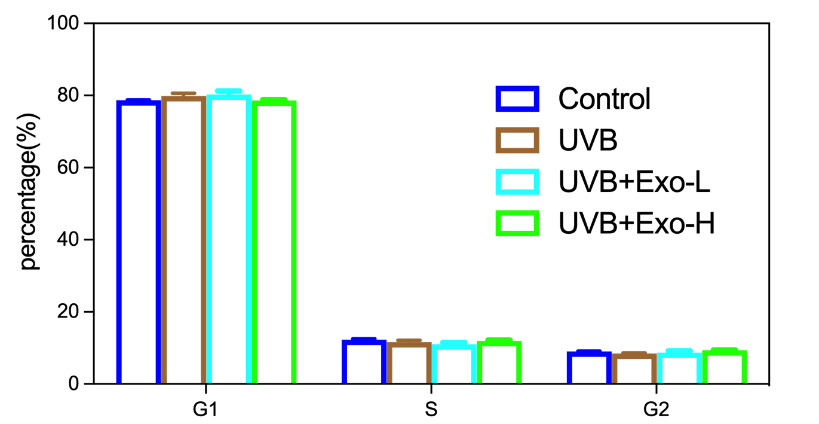


**Fig S3.** HFMSC-Exo has no significant effect on the cell cycle of HDF cells. (A) Representative flow cytometry images of the impact of HFMSC-Exo on HDF cell cycle. (B) The cell cycle statistical chart of Figure A.


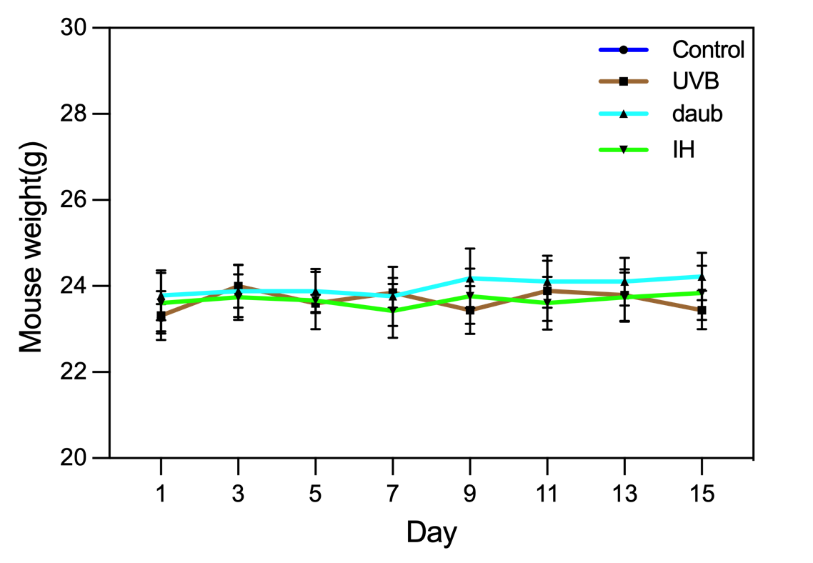


**Fig S4.** Weight changes in mouse during HFMSC-Exo treatment.


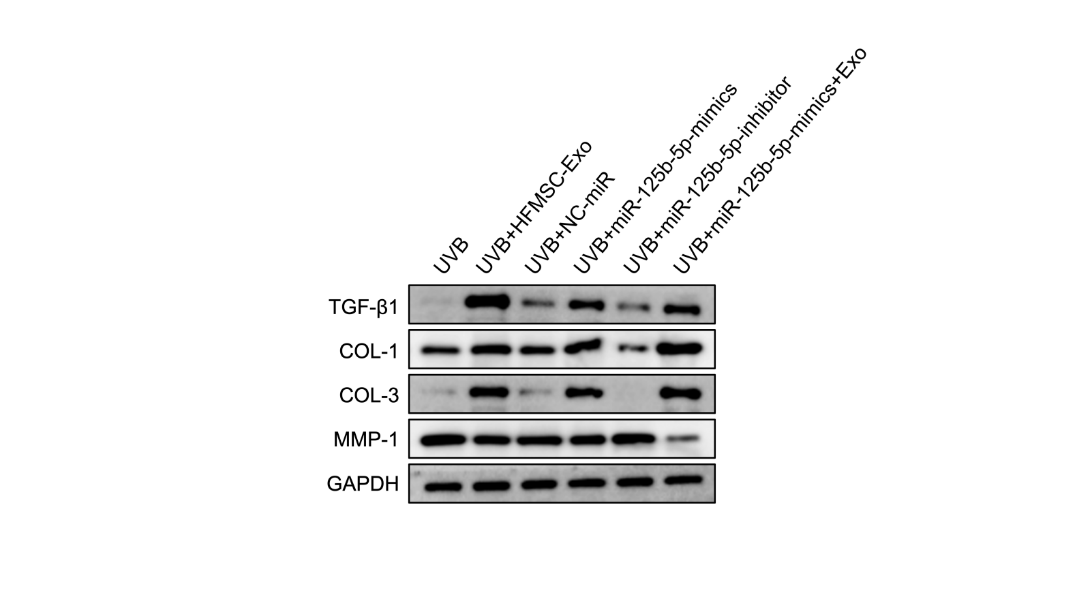


C

B

A


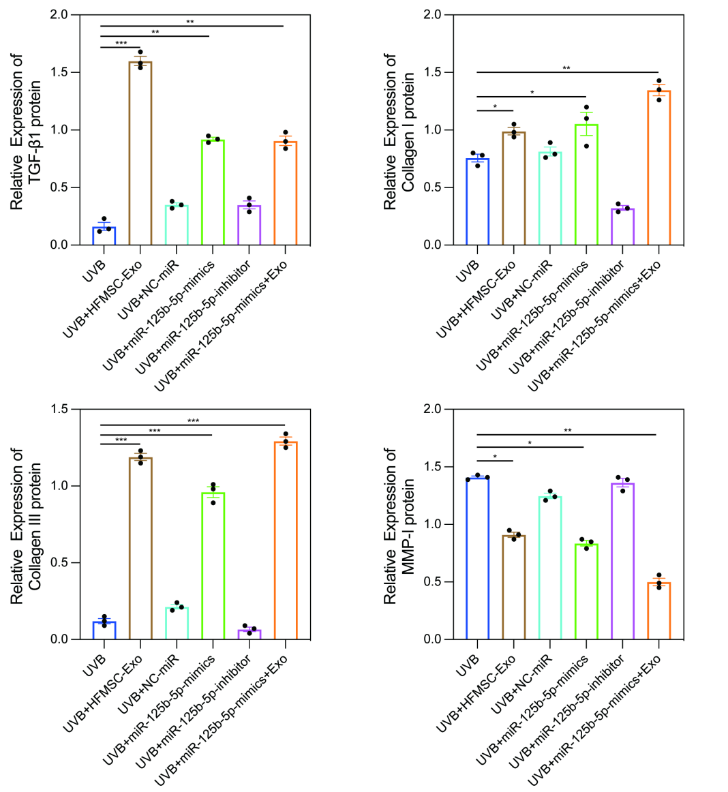


E

D

**Fig S5.** HFMSC-Exo can regulate the expression of COL-1, COL-3, and MMP-1 by regulating TGF-β1. (A) Representative WB images of TGF-β1, COL-1, COL-3, and MMP-1 proteins. (B,C,D&E) Statistical bar chart of protein expression in Figure A. *p<0.05，**p<0.01,***p<0.001.
